# Targeting the CHD3 chromatin remodeler exploits a synthetic lethal vulnerability in dual SMARCA4/SMARCA2-deficient cancers via derepression of PARD3B

**DOI:** 10.1038/s41698-026-01499-7

**Published:** 2026-05-22

**Authors:** Mariko Takeuchi, Yoshie Okimoto, Makoto Fukushima, Harumi Hirano, Hideaki Ogiwara

**Affiliations:** Division of Cancer Therapeutics, National Cancer Center Research Institute, Tokyo, Japan

**Keywords:** Cancer, Cell biology, Oncology

## Abstract

SWI/SNF-deficient cancers characterized by dual SMARCA4/SMARCA2 loss, including subsets of lung adenocarcinoma and small cell carcinoma of the ovary, hypercalcemic type, represent a major therapeutic challenge. While SMARCA2 paralog-targeting strategies exist for single-subunit loss of SMARCA4, dual SMARCA4/SMARCA2-deficient tumors lack actionable targets. Here, we identify the CHD3/NuRD nucleosome remodeling complex as a critical synthetic lethal vulnerability in these malignancies. Through integrated genomic analyses, we demonstrate that in the absence of SWI/SNF, CHD3 acts as an essential “epigenetic brake” specifically at the enhancer of the cell polarity regulator *PARD3B*. Loss of CHD3 triggers aberrant chromatin hyper-accessibility and toxic derepression of *PARD3B*. We further elucidate that *PARD3B* accumulation is a critical mediator of cell death, which is strongly associated with the attenuation of MYC signaling signatures. This attenuation likely arises from the spatial perturbation of upstream signaling hubs or secondary cellular responses, reducing MYC transcriptional output without degrading the protein itself. Therapeutically, CHD3 depletion led to robust tumor regression in dual SMARCA4/SMARCA2-deficient xenografts, validating the in vivo mechanism of PARD3B upregulation. Collectively, our study defines a novel mode of synthetic lethality driven by “gain-of-toxicity” rather than the loss of survival signals, uncovering a fatal cross-complex dependency. We propose that targeting CHD3 to trigger toxic PARD3B derepression offers a promising therapeutic avenue for treatment-refractory dual SMARCA4/SMARCA2-deficient cancers.

## Introduction

Mutations in genes encoding the SWI/SNF (BAF) chromatin remodeling complex are identified in over 20% of human cancers, constituting a major class of oncogenic drivers^1^. Among these, “SWI/SNF-deficient cancers,” characterized by the dual loss of the ATPase subunit SMARCA4 (BRG1) and its paralog SMARCA2 (BRM), or the complete loss of core subunits, encompass highly aggressive malignancies such as a subset of lung adenocarcinomas, small cell carcinoma of the ovary, hypercalcemic type (SCCOHT), thoracic SMARCA4-deficient undifferentiated tumors, and malignant rhabdoid tumors^2–8^. These malignancies maintain an undifferentiated state due to profound epigenetic dysregulation and are largely refractory to conventional chemotherapies^9,10^. Consequently, the establishment of novel therapeutic strategies targeting specific vulnerabilities arising from SWI/SNF deficiency remains an urgent clinical priority^9,10^.

To address this challenge, extensive efforts have been made to identify therapeutic targets exploiting the concept of synthetic lethality. To date, promising strategies include the inhibition of the antagonistic methyltransferase EZH2^11^ and the degradation or inhibition of the residual paralog SMARCA2^6,12,13^. Recently, we also reported the efficacy of inhibiting the histone acetyltransferases CBP/p300 in specific SWI/SNF-deficient contexts^14,15^. However, a critical limitation exists: therapeutic strategies targeting SMARCA2, while effective in SMARCA4-single-deficient cancers, are inherently inapplicable to dual SMARCA4/SMARCA2-deficient malignancies that lack the target paralog entirely.

Chromatin architecture is maintained through the coordinated action of diverse ATP-dependent remodeling complexes, including not only SWI/SNF but also ISWI, CHD, and INO80 families^16^. Whether cancer cells that have completely lost SWI/SNF—the primary regulatory machinery—acquire a lethal dependency on these “alternative chromatin remodeling complexes” to maintain genomic homeostasis remains largely unexplored, and the molecular mechanisms underlying such potential dependencies are unknown.

Chromodomain helicase DNA binding protein 3 (CHD3) is a core ATPase subunit of the nucleosome remodeling and deacetylase (NuRD) complex, forming a unique assembly that couples ATP-dependent nucleosome remodeling with histone deacetylation activities^17,18^. Although the NuRD complex has traditionally been viewed as a transcriptional repressor, recent studies suggest a context-dependent role in transcriptional activation, indicating functional pleiotropy^19^. However, the specific role of CHD3 in supporting cancer cell survival within the aberrant chromatin landscape of SWI/SNF deficiency remains entirely unelucidated.

In this study, through a targeted screen of chromatin remodeling factors in dual SMARCA4/SMARCA2-deficient cancer cells, we identified CHD3 as a novel synthetic lethal target. We demonstrate that in the absence of SWI/SNF, CHD3 functions as an essential “epigenetic brake” that maintains chromatin compaction at the locus of the cell polarity regulator *PARD3B*. Strikingly, we reveal that CHD3 loss triggers aberrant hyper-accessibility and transcriptional derepression of the *PARD3B* enhancer. The resulting toxic accumulation of PARD3B serves as a critical mediator of cell death, which is associated with the attenuation of MYC signaling signatures. This study elucidates a fatal interdependency between distinct chromatin remodeling complexes and provides in vivo proof-of-concept for CHD3-targeted therapy as a promising strategy for treatment-refractory dual SMARCA4/SMARCA2-deficient malignancies.

## Results

### Targeted screening identifies CHD3 as a prominent synthetic lethal vulnerability in dual SMARCA4/SMARCA2-deficient cancers

**Fig. 1:**
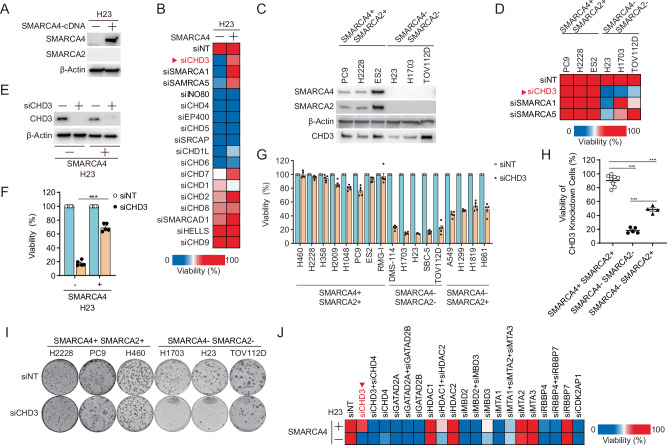
Targeted screening identifies CHD3 as a prominent synthetic lethal vulnerability in dual SMARCA4/SMARCA2-deficient cancers. **A** Immunoblot analysis of SMARCA4 and SMARCA2 expression in the isogenic model established from dual SMARCA4/SMARCA2-deficient H23 lung cancer cells and *SMARCA4*-cDNA restored H23 cells. β-Actin served as a loading control. **B** Heatmap showing the results of an siRNA screen targeting 17 ATP-dependent chromatin remodeling factors (excluding SWI/SNF subunits) in H23 cells. The screening was performed using SMARTpool siRNAs (a mixture of four distinct siRNAs per target gene) to minimize off-target effects. Cell viability of the H23 isogenic pair was assessed 7 days following a target gene knockdown. Data represent the mean viability from biological triplicates (*n* = 3), normalized to the non-targeting control (siNT). Blue indicates reduced cell viability. *CHD3, SMARCA1*, and *SMARCA5* were identified as top candidates. **C** Immunoblot characterization of a cell line panel comprising SMARCA4/SMARCA2-proficient (SMARCA4+/SMARCA2+) and dual SMARCA4/SMARCA2-deficient (SMARCA4−/SMARCA2−) lung and ovarian cancer lines. **D** Heatmap depicting the relative viability of SMARCA4/SMARCA2-proficient and dual SMARCA4/SMARCA2-deficient cell lines treated with siRNAs targeting the candidates identified in (**B**) and assessed 7 days post-transfection. Data represent the mean viability from biological triplicates (*n* = 3), normalized to the non-targeting control (siNT). Note that *CHD3* depletion selectively impacts dual SMARCA4/SMARCA2-deficient lines, whereas *SMARCA1* and *SMARCA5* depletion exhibit general toxicity. **E** Immunoblot validation of CHD3 knockdown efficiency in the H23 isogenic pair treated with control (siNT) or *CHD3*-targeting siRNA (siCHD3) for 96 h. **F** Cell viability of the H23 isogenic pair was assessed 7 days following *CHD3* knockdown. Data are normalized to siNT. Data are mean ± SD (*n* = 5). **G** Cell viability profiles of the expanded cell line panel were assessed 7 days after *CHD3* knockdown. Cells are grouped by SWI/SNF mutation status. Data are mean ± SD (*n* = 3). **H** Dot plot summarizing the viability of CHD3-depleted cells from (**G**). Note that dual SMARCA4/SMARCA2-deficient cells (SMARCA4−/SMARCA2−) show significantly higher sensitivity compared to proficient and single-deficient cells. Data are mean ± SD (SMARCA4+/SMARCA2+: *n* = 8, SMARCA4−/SMARCA2−: *n* = 5, SMARCA4−/SMARCA2+: *n* = 4; n indicates the number of cell lines per group). **I** Representative crystal violet staining images of the cell line panel treated with siNT or si*CHD3* and cultured for 10–14 days. **J** Heatmap showing the relative viability of H23 cells assessed 7 days following knockdown of individual NuRD complex subunits. Data represent the mean viability from biological triplicates (*n* = 3), normalized to the non-targeting control (siNT). *P < 0.05, **P < 0.01, ***P < 0.001 (Student’s *t*-test). See also Supplementary Fig. S1.

To identify epigenetic vulnerabilities in SWI/SNF-deficient cancers, particularly those harboring dual deficiency of SMARCA4 and SMARCA2, we established an isogenic cell model using the dual SMARCA4/SMARCA2-deficient H23 lung cancer cell line and its SMARCA4-restored counterpart (Fig. 1A). Immunoblot analysis confirmed that exogenous SMARCA4 restoration did not affect the inherent silencing of *SMARCA2* in these cells. We performed a focused siRNA screen targeting 17 ATP-dependent chromatin remodeling factors outside the SWI/SNF complex using SMARTpool siRNAs. Each pool comprised four distinct siRNA sequences targeting the same gene, an approach specifically utilized to maximize on-target knockdown efficiency while minimizing off-target effects. This targeted approach identified CHD3, SMARCA1, and SMARCA5 as top candidates exhibiting significant lethality in dual SMARCA4/SMARCA2-deficient cells (Fig. 1B). Next, we validated these candidates using SMARCA4/SMARCA2-proficient (PC9, H2228, ES-2) and dual SMARCA4/SMARCA2-deficient (H23, H1703, TOV112D) cell lines (Fig. 1C). While depletion of SMARCA1 and SMARCA5 exhibited no toxicity toward SWI/SNF-proficient cells, depletion of CHD3, a core ATPase of the NuRD complex, induced selective lethality in SMARCA4/SMARCA2-deficient lines without affecting proficient lines (Fig. 1D).

To further verify this selective dependency, we utilized the H23 isogenic model. Knockdown of CHD3 in the parental SMARCA4/SMARCA2-deficient H23 cells markedly reduced cell viability, whereas this lethality was significantly rescued in SMARCA4-restored H23 cells (Fig. 1E and F; Expression data in Supplementary Fig. S1A). To generalize these findings, we assembled a broad panel of cell lines comprising SMARCA4/SMARCA2-proficient (SMARCA4+/SMARCA2+: *n* = 8), dual SMARCA4/SMARCA2-deficient (SMARCA4−/SMARCA2−: *n* = 5), and single SMARCA4-deficient (SMARCA4−/SMARCA2+: *n* = 4) lines to evaluate CHD3 dependency (Fig. 1G, Supplementary Fig. S1B, Supplementary Table S1). CHD3 knockdown had minimal effect on the survival of proficient lines but significantly reduced the viability of dual SMARCA4/SMARCA2-deficient lines (Fig. 1H and I, Supplementary Fig. S1C and D). Interestingly, while survival of CHD3 knockdown in single SMARCA4-deficient lines was significantly reduced compared to proficient lines, its effect was intermediate compared to dual SMARCA4/SMARCA2-deficient lines (Fig. 1H). These results suggest that dual deficiency of SMARCA4 and SMARCA2, rather than SMARCA4 loss alone, is a critical determinant of synthetic lethal dependency on CHD3.

We confirmed the robustness of CHD3 dependency using distinct siRNA sequences, which reproduced specific lethality in dual SMARCA4/SMARCA2-deficient lines (H1703, H23, SBC-5) while sparing proficient cells (H460) (Supplementary Fig. S1E and F). Similarly, sustained CHD3 knockdown using a doxycycline-inducible shRNA system showed minimal effects on proficient H460 cells (Supplementary Fig. S1G and H) but induced progressive and significant viability loss in dual SMARCA4/SMARCA2-deficient H23 cells (Supplementary Fig. S1I and J).

In the H23 isogenic model, SMARCA4 restoration resulted in a slight decrease in *CHD3* mRNA levels (Supplementary Fig. S1K); however, across the cell line panel, no clear correlation was observed between SWI/SNF mutation status and *CHD3* expression levels (Supplementary Fig. S1L). This suggests that the dependency of dual SMARCA4/SMARCA2-deficient cancers on CHD3 reflects a functional addiction rather than mere overexpression.

Finally, to determine whether this synthetic lethal interaction is specific to CHD3 within the NuRD complex, we screened other NuRD subunits in H23 cells. Depletion of subunits other than CHD3 (e.g., MBD2, MTA families) did not elicit potent and selective lethal effects (Fig. 1J). Collectively, these data indicate that among the targeted chromatin remodelers, dual SMARCA4/SMARCA2-deficient cancers possess a prominent and fatal dependency on the CHD3 remodeling ATPase, particularly among NuRD complex components.

### Derepression of PARD3B drives CHD3 synthetic lethality

**Fig. 2:**
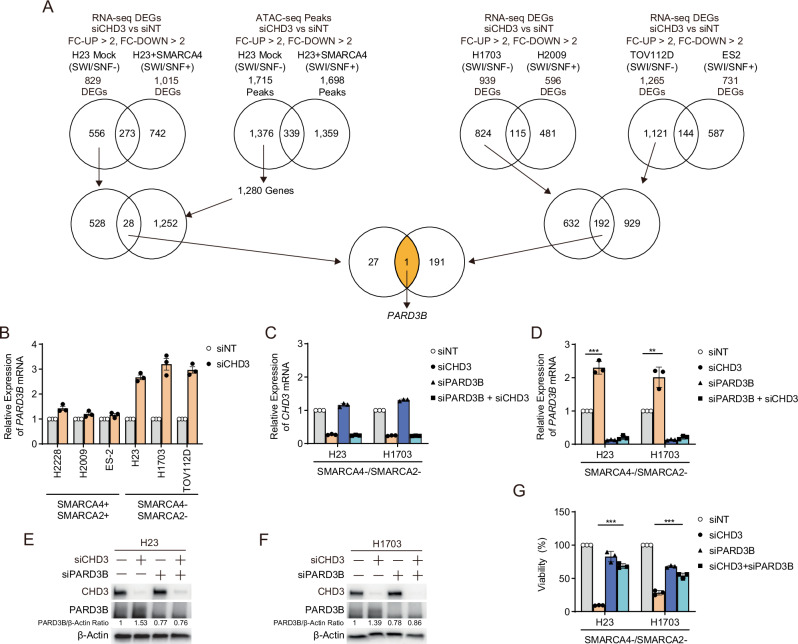
Derepression of PARD3B drives CHD3 synthetic lethality. **A** Integrated identification of synthetic lethal mediators. (**Left**) Venn diagrams summarizing the number of differentially expressed genes (RNA-seq) and altered chromatin peaks (ATAC-seq) in the H23 isogenic model upon *CHD3* knockdown. Genes and peaks specifically altered in SWI/SNF-deficient H23 Mock cells were intersected. (**Right**) RNA-seq analysis of lung (H1703 vs. H2009) and ovarian (TOV112D vs. ES-2) cancer cell lines. Overlap analysis identified *PARD3B* as the sole common candidate gene upregulated specifically in SWI/SNF-deficient cells upon *CHD3* depletion. **B** RT-qPCR analysis of *PARD3B* mRNA levels in SWI/SNF-proficient and -deficient cell lines treated for 48 h with control (siNT) or *CHD3*-targeting siRNA (siCHD3). Note the robust induction of *PARD3B* specifically in deficient lines. Data are mean ± SD (*n* = 3). RT-qPCR analysis of *CHD3* (**C**) and *PARD3B* (**D**) mRNA levels in SWI/SNF-deficient lung cancer cell lines (H23, H1703) treated for 48 h with siNT, siCHD3, *PARD3B*-targeting siRNA (siPARD3B), or the combination (siCHD3 + siPARD3B). Data are mean ± SD (*n* = 3). Immunoblot analysis of CHD3 and PARD3B protein levels in SWI/SNF-deficient lung cancer cell lines, H23 (**E**) and H1703 (**F**), following transfection with the indicated siRNAs for 96 h. β-Actin was used as a loading control. Densitometric quantification of PARD3B protein bands was performed using ImageJ software. The values indicated below the PARD3B blots represent the relative PARD3B/β-Actin signal ratios, normalized to the control lane (siCHD3[−]/siPARD3B[−]), which was set to 1. **G** Cell viability of H23 and H1703 cells treated as indicated for 7 days. Simultaneous knockdown of *PARD3B* significantly rescues the lethality induced by *CHD3* loss. Data are mean ± SD (*n* = 3). *P < 0.05, **P < 0.01, ***P < 0.001 (Student’s *t*-test). See also Supplementary Fig. S2.

As a core component of the NuRD chromatin remodeling complex, CHD3 regulates the transcription of various genes. We hypothesized that in dual SWI/SNF-deficient cancers, CHD3 suppression induces synthetic lethality by aberrantly upregulating specific CHD3 target genes. To identify the causative gene underlying this synthetic lethality, we employed an integrated analysis combining RNA sequencing (RNA-seq) for comprehensive gene expression profiling and ATAC sequencing (ATAC-seq) for chromatin accessibility profiling (Fig. 2A, left panel).

First, using the SMARCA4/SMARCA2-deficient H23 isogenic cell model, we identified differentially expressed genes (DEGs) upon CHD3 knockdown. We detected 829 DEGs in H23 Mock (SMARCA4-deficient) cells and 1015 DEGs in H23 + SMARCA4 (restored) cells (fold change > 2). Among these, 556 genes were differentially expressed specifically in H23 Mock cells. In parallel, ATAC-seq analysis identified 1715 and 1698 altered chromatin peaks (fold change > 2) in H23 Mock and H23+ SMARCA4 cells, respectively. Notably, 1376 peaks were specifically altered in H23 Mock cells, corresponding to 1280 genes located in the vicinity of these peaks. By intersecting mock-specific DEGs with genes exhibiting mock-specific chromatin alterations, we narrowed the candidates to 28 genes that showed transcriptional dysregulation potentially driven by chromatin structural changes in their regulatory regions (Supplementary Table S2). Subsequent gene ontology (GO) enrichment analysis of these 28 genes did not reveal any significantly enriched, coordinated biological pathways, suggesting context-specific heterogeneity rather than a single unified cascade.

To further refine these candidates for universality across cancer types and identify the core mediator, we performed RNA-seq analysis on panels of lung and ovarian cancer cell lines (Fig. 2A, Right panel). Comparing dual SMARCA4/SMARCA2-deficient lung cancer cells (H1703) with SMARCA4/SMARCA2-proficient cells (H2009), we identified 939 and 596 DEGs, respectively, with 824 genes specifically altered in the deficient line. Similarly, in ovarian cancer models, comparing dual SMARCA4/SMARCA2-deficient cells (TOV112D) with SMARCA4/SMARCA2-proficient cells (ES-2) revealed 1265 and 731 DEGs, respectively, with 1121 genes specifically altered in the deficient line. Intersection of the deficiency-specific DEGs from both lung and ovarian models yielded 192 common genes.

Strikingly, *PARD3B* was identified as the sole candidate gene shared between the 28 genes from the isogenic model and the 192 genes from the cell line panels. PARD3B is a scaffold protein known to regulate cell polarity and tight junction formation, although its role in cancer cell survival is context-dependent^20^. Validation by RT-qPCR confirmed that robust induction of *PARD3B* mRNA upon CHD3 depletion occurred specifically in dual SMARCA4/SMARCA2-deficient cell lines (Fig. 2B).

To determine whether PARD3B induction is the direct driver of synthetic lethality in SWI/SNF-deficient cells, we performed rescue experiments using simultaneous knockdown. In dual SMARCA4/SMARCA2-deficient lung (H23, H1703) and ovarian (TOV112D) cancer cell lines, CHD3 knockdown reduced *CHD3* mRNA (Fig. 2C, Supplementary Fig. S2A) and led to a marked increase in *PARD3B* mRNA (Fig. 2D, Supplementary Fig. S2B). Furthermore, densitometric quantification of immunoblots confirmed a consistent 1.4- to 1.5-fold increase in endogenous PARD3B protein levels following CHD3 knockdown (Fig. 2E and F), resulting in significant cell viability loss (Fig. 2G, Supplementary Fig. S2C). However, simultaneous knockdown of CHD3 and PARD3B significantly, and in some cases remarkably, rescued the lethality observed with CHD3 single knockdown (Fig. 2G, Supplementary Fig. S2C). These results demonstrate that the derepression of PARD3B is a critical downstream mediator driving the observed synthetic lethality, although we cannot entirely exclude the potential contributions of other factors affected by CHD3 loss.

Given that the CHD3/NuRD complex contains histone deacetylases (HDAC1 and HDAC2) as core subunits, we investigated whether deacetylase activity is required for CHD3-mediated repression of *PARD3B*. We treated dual SMARCA4/SMARCA2-deficient cell lines (H23, H1703, and TOV112D) with the pan-HDAC inhibitor Vorinostat. The response was heterogeneous: PARD3B expression increased in TOV112D cells, decreased in H1703 cells, and remained unchanged in H23 cells (Supplementary Fig. S2D). These findings suggest that the repressive function of CHD3 at the *PARD3B* locus is not solely dependent on HDAC1/2 enzymatic activity, implying that the structural integrity or nucleosome remodeling capacity of the NuRD complex is critical for this regulation.

To further investigate the clinical relevance of the CHD3–PARD3B epigenetic axis, we analyzed PARD3B expression in primary human tumors using the TCGA PanCancer Atlas Lung Adenocarcinoma dataset (*n* = 510). Because evaluating *SMARCA2* epigenetic silencing via bulk RNA-seq is inherently confounded by normal stromal cell expression, we utilized *SMARCA4* mutation status—which highly correlates with concurrent *SMARCA2* silencing in lung adenocarcinoma—as a robust proxy for SWI/SNF deficiency^6,8,21^. According to our “epigenetic brake” model, we hypothesized that in actual patient tumors harboring *SMARCA4* mutations, the intact CHD3–NuRD complex successfully suppresses the toxic derepression of *PARD3B*, thereby allowing these SWI/SNF-deficient tumors to survive. Perfectly consistent with this hypothesis, we found that *PARD3B* mRNA expression was not elevated in *SMARCA4*-mutated tumors; in fact, its basal expression was maintained at a statistically significantly lower level compared to *SMARCA4*-wild-type tumors (*P* < 0.05) (Supplementary Fig. S2E). This clinical observation strongly supports the premise that SWI/SNF-deficient cancers inherently rely on the repressive function of CHD3 to keep *PARD3B* expression below a toxic threshold in vivo.

### CHD3 functions as an epigenetic brake specifically at the *PARD3B* enhancer

**Fig. 3:**
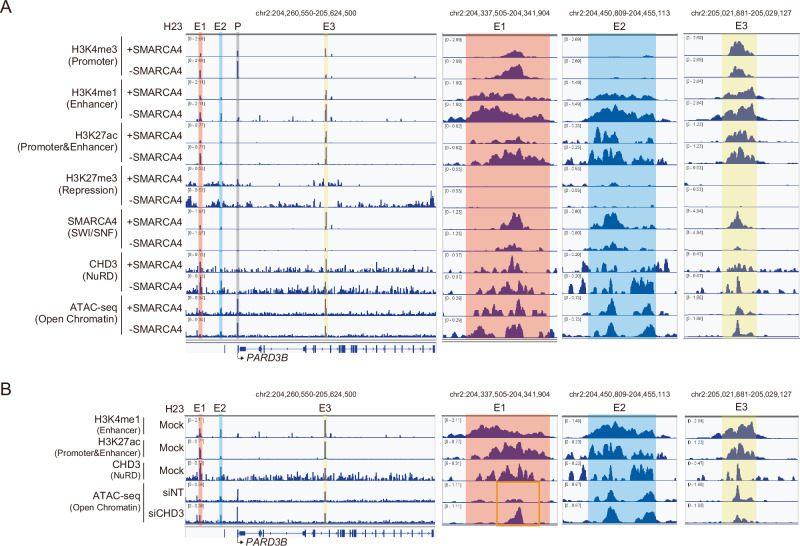
CHD3 maintains chromatin compaction at the *PARD3B* enhancer in dual SMARCA4/SMARCA2-deficient cells. **A** Genome browser tracks showing the occupancy of H3K4me3, H3K4me1, H3K27ac, H3K27me3, SMARCA4, and CHD3, along with ATAC-seq signals at the *PARD3B* locus in the H23 isogenic model. P denotes putative promoter region. E1, E2, and E3 denote putative enhancer regions where SMARCA4 and CHD3 co-localize. Note that CHD3 binding is retained in SWI/SNF-deficient (-SMARCA4) cells. **B** Genome browser view of the *PARD3B* locus. The top three tracks display baseline CUT&RUN-seq profiles (H3K4me1, H3K27ac, and CHD3) in H23 Mock cells (SMARCA4-deficient). These tracks establish the basal enhancer landscape and endogenous CHD3 occupancy prior to any siRNA perturbation. The bottom two tracks display ATAC-seq signals, illustrating changes in chromatin accessibility following transfection with non-targeting control (siNT) or *CHD3*-targeting (siCHD3) siRNAs for 96 h. Magnified views of the putative enhancer regions (E1, E2, and E3) are shown on the right. Note the prominent increase in chromatin accessibility specifically at the E1 region upon *CHD3* depletion (highlighted by the orange box). See also Supplementary Fig. S3.

We next investigated how CHD3 regulates *PARD3B* expression. In SMARCA4-restored H23 cells, CUT&RUN sequencing revealed that CHD3 occupies both promoter (co-localizing with H3K4me3/H3K27ac) and enhancer (co-localizing with H3K4me1/H3K27ac) regions genome-wide (Supplementary Fig. S3A). Notably, CHD3 exhibited a strong tendency to co-localize with SMARCA4, particularly at enhancer regions (Supplementary Fig. S3A). While these global chromatin profiling data suggest a broad functional interplay, we focused our detailed mechanistic investigation strictly on the *PARD3B* locus as the primary mediator of the synthetic lethal phenotype. Detailed inspection of the *PARD3B* locus in SMARCA4-restored H23 cells revealed that CHD3 and SMARCA4 co-localize at multiple putative enhancer regions (E1, E2, E3) rather than the promoter region (P) (Fig. 3A). Importantly, CHD3 occupancy at these regions was maintained even in the absence of SMARCA4 (H23 Mock). To assess chromatin accessibility, we performed ATAC-seq. In dual SMARCA4/SMARCA2-deficient H23 cells, CHD3 knockdown led to a marked increase in chromatin accessibility (ATAC signal) at *PARD3B* enhancers, most prominently at the E1 region located on the 5’ side of the gene (Fig. 3B). Since our primary objective was to define the regulatory elements driving the transcriptional upregulation (derepression) of *PARD3B*, we focused our interpretation on the E1 enhancer, which logically correlated with this phenotype by showing increased accessibility. Similarly, in other dual SMARCA4/SMARCA2-deficient cells (H1703, TOV112D), CHD3 depletion induced a significant gain of chromatin accessibility at the 5’ region of the *PARD3B* locus (Supplementary Fig. S3B). These findings indicate that in the absence of SWI/SNF, CHD3 acts as an essential “epigenetic brake” to maintain a closed chromatin state specifically at the *PARD3B* locus, preventing its transcriptional derepression.

### PARD3B accumulation is associated with the attenuation of *MYC* signaling signatures

**Fig. 4:**
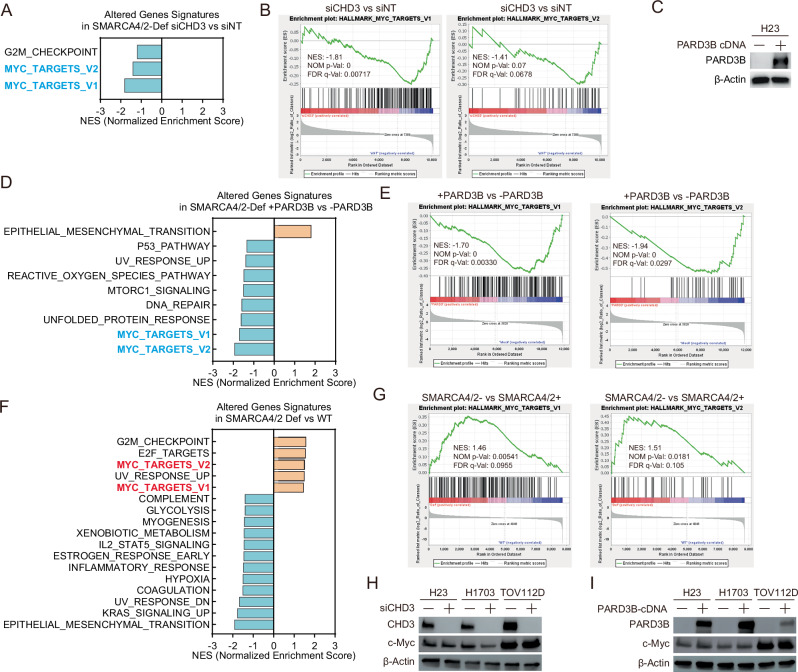
PARD3B accumulation is associated with the attenuation of *MYC* signaling signatures. **A** Normalized enrichment scores (NES) of hallmark gene sets were significantly altered in H23 cells upon *CHD3* knockdown (siCHD3) compared to control (siNT). Note the suppression of MYC target signatures. **B** Gene Set Enrichment Analysis (GSEA) plots showing significant suppression of *HALLMARK_MYC_TARGETS_V2* and *V1* signatures in H23 cells upon *CHD3* knockdown (siCHD3) compared to control (siNT). **C** Immunoblot confirmation of PARD3B overexpression (OE) in H23 cells transfected with a *PARD3B*-cDNA vector. **D** Normalized enrichment scores (NES) of hallmark gene sets were significantly altered in H23 cells upon PARD3B OE. Note the suppression of *MYC* target signatures. **E** GSEA plots showing suppression of *HALLMARK_MYC_TARGETS_V1* (left) and *V2* (right) signatures in H23 cells upon PARD3B OE. **F** NES of hallmark gene sets is significantly enriched in dual SMARCA4/SMARCA2-deficient cells (H1703) compared to SMARCA4/SMARCA2-proficient cells (H2009). MYC target signatures are among the top upregulated pathways. **G** GSEA plots showing enrichment of *HALLMARK_MYC_TARGETS_V1* (left) and *V2* (right) signatures in dual SMARCA4/SMARCA2-deficient cells compared to SMARCA4/SMARCA2-proficient cells. Immunoblot analysis of c-MYC protein in dual SMARCA4/SMARCA2-deficient cell lines (H23, H1703, TOV112D) treated for 96 h with siNT vs. siCHD3 (**H**) or Mock vs. *PARD3B*-cDNA (**I**). Paradoxically, c-MYC protein levels remain stable despite the transcriptional suppression of its targets. Statistical significance for GSEA was defined as Nominal *p*-value < 0.05 and FDR *q*-value < 0.25. Immunoblots are representative of at least two independent experiments. See also Supplementary Fig. S4.

To investigate the mechanisms by which CHD3 suppression and PARD3B overexpression induce cell death in SWI/SNF-deficient cells, we sought to identify common molecular pathways enriched in the altered gene signatures. Gene Set Enrichment Analysis (GSEA) revealed that *HALLMARK_MYC_TARGETS_V1* and *V2* signatures were commonly suppressed by both CHD3 knockdown and PARD3B overexpression in dual SMARCA4/SMARCA2-deficient cells (Fig. 4A–E, Supplementary Fig. S4A–G). Conversely, comparisons between dual SMARCA4/SMARCA2-deficient and SMARCA4/SMARCA2-proficient cell lines revealed that MYC target signatures were significantly enriched in dual SMARCA4/SMARCA2-deficient cells (Fig. 4F and G, Supplementary Fig. S4H–J). This suggests that while dual SMARCA4/SMARCA2-deficient cells are inherently addicted to hyperactive MYC signaling, PARD3B accumulation following CHD3 suppression is strongly associated with the attenuation of these transcriptional programs. Here, we observed a paradoxical phenomenon. Immunoblot analysis revealed that neither CHD3 knockdown nor PARD3B overexpression caused a marked reduction in c-MYC protein levels in dual SMARCA4/SMARCA2-deficient cells (H23, H1703, TOV112D) (Fig. 4H and I).

To resolve this paradox and identify upstream mechanisms associated with these altered *MYC* signatures, we performed WikiPathway analysis. Unlike GSEA, which captures broad trends in gene expression, WikiPathway analysis is based on curated signal transduction maps, enabling the identification of specific signaling cascades. We identified *VEGFR2*-associated pathways as common targets downregulated by CHD3 suppression/PARD3B overexpression and upregulated in dual SMARCA4/SMARCA2-deficient cells (Supplementary Fig. S4K). VEGFR2 is known to regulate MYC via upstream PI3K-AKT and MAPK pathways. We therefore examined the phosphorylation status of major upstream effectors (VEGFR, AKT, ERK, STAT3). We found that while these pathways were partially suppressed in certain contexts, no specific kinase was uniformly inactivated across all cell lines upon CHD3 suppression or PARD3B overexpression (Supplementary Fig. S4L–O). This heterogeneity suggests that PARD3B accumulation does not inhibit a single kinase but rather spatially perturbs signaling hubs, leading to context-dependent attenuation of upstream signals.

Collectively, these data suggest that PARD3B accumulation following CHD3 suppression is associated with a reduction of signatures associated with *MYC* signaling—where its transcriptional output is reduced despite the presence of MYC protein. Although we cannot rule out the possibility that the reduction in *MYC* signatures is a secondary consequence of broader cellular perturbations, such as cell cycle arrest, this profound attenuation of MYC signaling correlates with the ultimate collapse of pro-survival signaling in dual SMARCA4/SMARCA2-deficient cells.

### Therapeutic exploitation of CHD3 dependency in dual SMARCA4/SMARCA2-deficient cancers in vivo

**Fig. 5:**
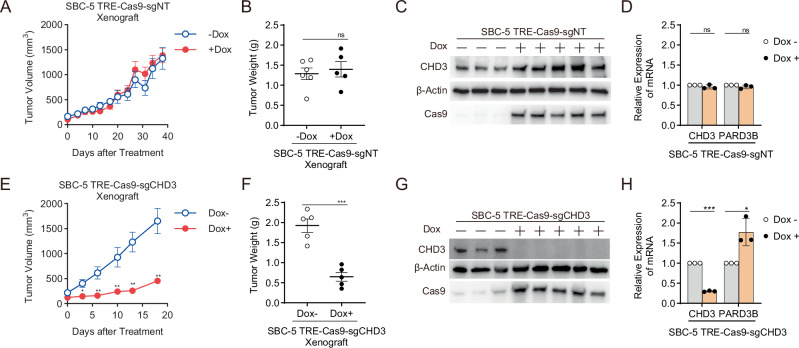
Therapeutic exploitation of CHD3 dependency in dual SMARCA4/SMARCA2-deficient cancers in vivo. Tumor growth curves (**A**) and final tumor weights (**B**) of SBC-5 xenografts expressing Dox-inducible Cas9 and non-targeting sgRNA (sgNT). Mice bearing palpable tumors were treated with vehicle (−Dox: *n* = 6) or Doxycycline (+Dox: *n* = 5). No significant differences were observed. Data are presented as mean ± SEM. ns, not significant. (two-tailed Student’s *t*-test). **C** Immunoblot analysis of CHD3 and Cas9 expression in sgNT tumors harvested at the endpoint. β-Actin served as a loading control. **D** Relative mRNA expression levels of *CHD3* and *PARD3B* in SBC-5-sgNT xenograft tumors treated with (Dox+) or without (Dox−) doxycycline. Data are presented as mean ± SD (*n* = 3). ns not significant (two-tailed Student’s *t*-test). Tumor growth curves (**E**) and final tumor weights (**F**) of SBC-5 xenografts expressing Dox-inducible Cas9 and *CHD3*-targeting sgRNA (sgCHD3). Mice bearing palpable tumors were treated with vehicle (−Dox: *n* = 5) or Doxycycline (+Dox: *n* = 5). Note the robust tumor regression induced by Dox treatment. Data are presented as mean ± SEM. *P < 0.05, **P < 0.01, ***P < 0.001 (Student’s *t*-test). (two-tailed Student’s *t*-test). **G** Immunoblot analysis of CHD3 and Cas9 expression in sgCHD3 tumors. Effective depletion of CHD3 protein upon Cas9 induction (+Dox) is confirmed. β-Actin served as a loading control. **H** Relative mRNA expression levels of *CHD3* and *PARD3B* in SBC-5-sgCHD3 xenograft tumors treated with or without doxycycline. Note the significant upregulation of *PARD3B* expression following *CHD3* depletion. Data are presented as mean ± SD (*n* = 3). *P < 0.05, **P < 0.01, ***P < 0.001 (two-tailed Student’s *t*-test).

Finally, we evaluated the therapeutic potential of targeting CHD3 in vivo. We utilized the SBC-5 cell line, which demonstrated the most robust tumor engraftment among the validated dual SMARCA4/SMARCA2-deficient models. We established cell line-derived xenograft (CDX) models using dual SMARCA4/SMARCA2-deficient SBC-5 cells engineered to express doxycycline (Dox)-inducible Cas9 and single-guide RNAs (sgRNAs) targeting either a non-targeting control (sgNT) or *CHD3* (sgCHD3). In the control SBC-5-TRE-Cas9-sgNT xenograft model, Dox administration induced Cas9 expression but did not affect CHD3 protein levels, and no significant difference in tumor growth was observed compared to the vehicle-treated group (Fig. 5A–C). Consistently, RT-qPCR analysis of tumor lysates confirmed that Dox treatment altered neither *CHD3* nor *PARD3B* mRNA expression levels (Fig. 5D).

In stark contrast, Dox administration in the SBC-5-TRE-Cas9-sgCHD3 xenograft model induced potent tumor regression compared to controls (Fig. 5E and F). Immunoblot analysis of tumor tissues confirmed the induction of Cas9 expression and the efficient depletion of CHD3 protein in Dox-treated tumors (Fig. 5G). Furthermore, validating our in vitro findings, RT-qPCR analysis of residual tumor tissues revealed a significant downregulation of *CHD3* concomitant with a significant upregulation of *PARD3B* (Fig. 5H). These results provide proof-of-concept that targeting CHD3 effectively exploits the epigenetic vulnerability of dual SMARCA4/SMARCA2-deficient cancers in vivo.

## Discussion

SWI/SNF-deficient cancers, particularly those characterized by the dual loss of SMARCA4 and SMARCA2, represent a therapeutic challenge due to their undifferentiated phenotype and lack of actionable drivers^2–5^. While current synthetic lethal strategies primarily exploit paralog dependencies—such as targeting SMARCA2 in SMARCA4-deficient tumors^6,12,13^—our study uncovers a distinct vulnerability: a fatal “cross-complex dependency” on the CHD3/NuRD complex. We demonstrate that the loss of the SWI/SNF complex, a master regulator of chromatin accessibility^1^, renders cancer cells strictly addicted to the opposing chromatin remodeler CHD3 to maintain epigenetic homeostasis. A notable distinction arising from our findings is the functional divergence between CHD3 and its closely related paralog, CHD4, within the NuRD complex. While extensive functional targeted screens indicate that CHD4 acts as a pan-essential gene required for basal cellular homeostasis irrespective of genetic context, CHD3 exhibits a highly selective synthetic lethality. This phenotypic specificity aligns with previous biochemical evidence demonstrating that CHD3 and CHD4 form mutually exclusive, compositionally distinct NuRD complexes characterized by different, albeit partially overlapping, genomic localizations and functional roles^22^. We postulate that whereas the CHD4–NuRD complex is globally required for fundamental chromatin maintenance, the CHD3–NuRD complex exerts specialized, context-dependent repressive functions at specific regulatory elements, such as the *PARD3B* enhancers. In SWI/SNF-proficient cells, redundant epigenetic networks likely compensate for the loss of CHD3. However, upon the inactivation of the master chromatin regulator SWI/SNF, cells lose this plasticity and become critically reliant on this specialized CHD3-mediated “epigenetic brake” to prevent the toxic derepression of lethal downstream targets. This finding expands the concept of synthetic lethality beyond gene families (paralogs) to inter-complex interactions, suggesting that the balance between distinct chromatin remodeling machineries is a critical determinant of cancer cell survival.

Recently, Schick et al. reported that the loss of SWI/SNF complex function is functionally compensated by another chromatin remodeler, the EP400/TIP60 complex^23^. Their study demonstrated that EP400 deficiency creates a synthetic lethal vulnerability to SWI/SNF inhibition, supporting the concept of functional interplay between distinct remodeling complexes. However, while their study highlighted that EP400 primarily maintains chromatin accessibility at gene promoters, our findings reveal that CHD3 plays a critical role predominantly at enhancer regions, such as the *PARD3B* enhancer. Indeed, in our screening, EP400 exhibited potent toxicity not only in SWI/SNF-deficient cells but also in proficient cells, limiting its selectivity as a therapeutic target in our context. In contrast, CHD3 inhibition demonstrated high selectivity for SWI/SNF-deficient cancers. This suggests that dependencies involving enhancer regulation (CHD3) may represent a more specific vulnerability in the context of SWI/SNF deficiency compared to those involving basal promoter functions (EP400).

A key conceptual advance of this study is the identification of “toxic derepression” as a driver of synthetic lethality. Unlike canonical synthetic lethal interactions that involve the loss of essential survival signals, we show that SWI/SNF-deficient cells succumb to death because they fail to repress a gene, *PARD3B*, in the absence of CHD3. Our data indicate that CHD3, a core ATPase of the NuRD complex known to couple chromatin remodeling with histone deacetylation^24^, functions as an essential “epigenetic brake” at the *PARD3B* enhancer. Interestingly, although CHD3 and SMARCA4 co-localize at multiple putative enhancer regions of the *PARD3B* locus (E1, E2, and E3), we observed that only the E1 region exhibited marked hyper-accessibility following CHD3 depletion. We hypothesize that this differential response stems from distinct, context-dependent epigenetic regulatory networks operating at these individual elements. The E1 region likely functions as a poised enhancer strictly dependent on the repressive activity of the CHD3–NuRD complex in the absence of SWI/SNF. In contrast, the chromatin state at the E2 and E3 regions remained closed, or even exhibited decreased accessibility (as observed at E3). This intriguing opposite consequence might result from the compensatory recruitment and binding of alternative epigenetic silencers or repressor complexes following CHD3 eviction. The precise molecular determinants dictating these enhancer-specific dependencies and dynamic repressor exchanges remain an intriguing area for future investigation. While NuRD complexes can function as transcriptional activators in certain contexts^22^, our findings underscore their critical role as a repressor in this setting. Importantly, while artificial overexpression of scaffolding proteins like PARD3B could be broadly toxic to various cell types due to non-specific signaling perturbations, the therapeutic vulnerability we identified relies strictly on its context-dependent endogenous derepression. In SWI/SNF-proficient cells, robust and redundant epigenetic mechanisms successfully maintain the *PARD3B* locus in a repressed state, rendering CHD3 dispensable. In the absence of SWI/SNF-mediated regulation, however, this CHD3-mediated brake becomes the sole fail-safe mechanism; its removal triggers the unleashed endogenous expression of PARD3B to toxic levels. This gain-of-toxicity model provides a clear mechanistic rationale for why CHD3 is dispensable in normal cells but essential in the context of SWI/SNF deficiency.

Furthermore, to understand how PARD3B accumulation contributes to cell death, we investigated its downstream functional associations. Through GSEA analysis, we observed a paradoxical phenomenon where MYC target signatures were significantly suppressed despite stable c-MYC protein levels. We propose that PARD3B accumulation is associated with a reduction of signatures associated with MYC signaling. PARD3B is a cell polarity regulator known to organize spatial signaling domains^20^. Its aberrant accumulation likely perturbs the spatial organization of upstream signaling hubs—such as PI3K/AKT and MAPK pathways—potentially leading to a context-dependent attenuation of kinase activities required for optimal MYC function. Importantly, we acknowledge that the precise molecular cascade linking PARD3B accumulation to MYC suppression remains incompletely resolved. As an alternative interpretation, we cannot rule out the possibility that the observed reduction in MYC signatures is a secondary consequence of broader cellular perturbations, such as cell cycle arrest or generalized cellular toxicity following *CHD3* depletion. Future studies incorporating detailed cell cycle analyses (e.g., FACS) and MYC chromatin occupancy profiling (e.g., CUT&RUN or ChIP-seq) will be required to definitively establish the direct relationship between PARD3B accumulation, cell cycle dynamics, and MYC transcriptional activity. Given that SWI/SNF-deficient cancers are highly dependent on MYC signaling, the coordinate downregulation of the entire MYC network—whether through spatial signaling perturbation or as an indirect result of broader cellular perturbations—is strongly correlated with compromised cell survival. Thus, while the exact downstream mechanisms warrant further investigation, the CHD3–PARD3B axis constitutes an epigenetic vulnerability that, when targeted, fatally disrupts the pro-survival state upon which these cancers rely.

Clinically, our findings offer a promising therapeutic avenue for the most aggressive subset of SWI/SNF-deficient malignancies. Cancers with dual SMARCA4/SMARCA2 deficiency, including undifferentiated thoracic sarcomas^3^ and a subset of lung adenocarcinomas^6^, are refractory to emerging therapies that target SMARCA2^6,12,13^, as they lack the paralog target entirely. Our study positions CHD3 as a critical target specifically for this “treatment-refractory” population. Importantly, our in vivo analysis confirmed that tumor regression driven by CHD3 depletion was accompanied by the marked upregulation of *PARD3B*, mirroring the “derepression” mechanism observed in vitro. By exploiting the cell’s reliance on CHD3 to suppress PARD3B toxicity, CHD3-targeted therapy could induce tumor regression in patients who currently have no effective treatment options. The translational significance of targeting this vulnerability is underscored by our analysis of clinical datasets. In the TCGA Lung Adenocarcinoma cohort, *PARD3B* expression remains firmly suppressed in *SMARCA4*-mutated patient tumors, confirming that the CHD3-mediated epigenetic brake is actively engaged and indispensable for tumor survival in vivo. Because these SWI/SNF-deficient tumors have lost the redundant chromatin remodeling plasticity present in normal cells, pharmacologically dismantling this final, critical CHD3 brake represents a highly rational and selective therapeutic strategy.

Regarding therapeutic modalities, our in vivo data demonstrate that depleting CHD3 protein using the CRISPR/Cas9 system is highly effective. Given that CHD3 functions as a structural component of the NuRD complex, simple enzymatic inhibition of its ATPase activity or associated HDAC activity might be insufficient to dislodge the complex from chromatin or release the epigenetic brake. Consistent with this, our screening revealed that the knockdown of HDAC1 and/or HDAC2 failed to induce synthetic lethality, and pharmacological HDAC inhibition using Vorinostat did not consistently induce *PARD3B* derepression. These findings strongly suggest that the structural integrity of the CHD3-containing complex, rather than its deacetylase activity alone, is critical for maintaining cell survival. Therefore, we propose that strategies inducing CHD3 protein degradation—such as Proteolysis Targeting Chimeras (PROTACs)^25^—represent the most viable approach for drug development.

It is important to note the limitations of our initial screening approach. Because our targeted siRNA screen was specifically focused on ATP-dependent chromatin remodeling factors rather than being genome-wide, we cannot conclude that CHD3 represents the exclusive or universally strongest vulnerability across the entire genome in SWI/SNF-deficient cancers. Rather, our findings establish CHD3 as a highly prominent synthetic lethal target within the restricted landscape of epigenetic regulators. Whether other non-epigenetic pathways or broader cellular networks harbor equally potent vulnerabilities remains an important area for future genome-wide functional genomics studies.

Although we focused on *PARD3B* as the universal mediator consistently upregulated across distinct cancer lineages (lung and ovarian) upon *CHD3* depletion, our integrated transcriptomic and epigenomic analyses in the H23 isogenic model initially identified 28 candidate genes showing significant alterations. We acknowledge it is highly plausible that, within specific cellular contexts, some of these other target genes might collaboratively contribute to the lethal phenotype or the perturbation of downstream signaling networks following *CHD3* loss. Further context-specific functional evaluations of these additional candidates remain an area for future investigation. Given the broad epigenetic regulatory roles of the NuRD complex, disruption of CHD3 inevitably induces widespread chromatin and transcriptional changes. Therefore, while our rescue experiments demonstrate that PARD3B derepression is a critical downstream mediator of the synthetic lethal phenotype, we acknowledge that it represents one of several downstream consequences of CHD3 depletion rather than the sole effector.

Furthermore, we acknowledge a limitation regarding the global generalization of our mechanistic findings. While our initial CUT&RUN analysis demonstrated that CHD3 widely co-localizes with SMARCA4 across the genome, particularly at enhancer regions, our detailed epigenetic and transcriptional investigation was strictly focused on the *PARD3B* locus. Therefore, we cannot definitively conclude that the specific “epigenetic brake” mechanism observed at the *PARD3B* enhancers universally applies to all other differentially expressed genes. To determine the global extent of this functional interplay—specifically, whether all genes exhibiting increased chromatin accessibility and transcriptional upregulation following CHD3 depletion share this exact regulatory mechanism—future comprehensive studies integrating genome-wide CUT&RUN, ATAC-seq, and RNA-seq analyses are required.

Conceptually, our findings advocate for expanding the framework of synthetic lethality beyond the traditional “one-to-one” interaction model, where a single genetic alteration sensitizes cells to the inhibition of a single target. We propose that “next-generation” synthetic lethality involves higher-order relationships among three or more factors. Previously, we established a “one-to-two” synthetic lethal strategy, termed dual paralog inhibition, where a single genetic loss (e.g., SMARCB1 deficiency) creates a dependency on the simultaneous inhibition of two paralogous factors (e.g., CBP and p300)^14,15^. In contrast, the present study highlights a “two-to-one” model in which the dual deficiency of two genes (SMARCA4 and SMARCA2) converges to create a critical dependency on a single distinct factor (CHD3). These findings collectively suggest that cancer vulnerabilities are not limited to binary interactions but rather emerge from complex, multi-gene network imbalances. Therefore, future therapeutic strategies should focus on identifying and exploiting these “one-to-two” or “two-to-one” multi-factor relationships to broaden the scope of precision medicine for refractory cancers.

## Methods

### Animal ethics statement

All mouse experiments were approved by the National Cancer Center Animal Ethics Committee (Protocol Number: T19-013-M06) and were performed in accordance with the *Act on Welfare and Management of Animals* and the *Standards for Care and Keeping and Reducing Pain of Laboratory Animals*. Mice were monitored for clinical signs, tumor volume, and body weight as specified in the experimental protocols. Tumor implantation and measurements were performed without the use of anesthesia, in accordance with the approved institutional protocols. At the experimental endpoints, animals were euthanized by CO_2_ asphyxiation. All procedures were performed before reaching the approved humane endpoints, defined as a tumor volume of 2000 mm^3^ or a body weight loss of 20% or the appearance of overt clinical signs of pain or distress. The maximum tumor burden was not exceeded in any of the experiments.

### Cell Lines and culture conditions

Cells were maintained at 37 °C in a humidified incubator containing 5% CO_2_. The culture medium comprised DMEM/F-12 (Wako, 048-29785) supplemented with 10% fetal bovine serum (FBS; Gibco/Life Technologies), 10% GlutaMAX Supplement (Gibco, 41550021), 100 U/mL penicillin, and 100 μg/mL streptomycin (Wako, 168-23191). Human lung cancer cell lines including NCI-H23 (RRID:CVCL_1547), NCI-H1703 (RRID:CVCL_1490), NCI-H2009 (RRID:CVCL_1514), NCI-H460 (RRID:CVCL_0459), NCI-H2228 (RRID:CVCL_1543), NCI-H358 (RRID:CVCL_1559), NCI-H1048 (RRID:CVCL_1453), PC-9 (RRID:CVCL_B260), DMS-114 (RRID:CVCL_1174), SBC-5 (RRID:CVCL_1679), A549 (RRID:CVCL_0023), NCI-H1299 (RRID:CVCL_0060), NCI-H1819 (RRID:CVCL_1497), and NCI-H661 (RRID:CVCL_1577), and ovarian cancer cell lines including TOV-112D (RRID:CVCL_3612), ES-2 (RRID:CVCL_3509), and RMG-I (RRID:CVCL_1662) were used in this study. NCI-H23, NCI-H1703, NCI-H2009, NCI-H460, NCI-H2228, NCI-H358, NCI-H1048, DMS-114, A549, NCI-H1299, NCI-H1819, NCI-H661, TOV-112D, and ES-2 were obtained from the American Type Culture Collection (ATCC). PC-9 was obtained from Immuno-Biological Laboratories (IBL). SBC-5 and RMG-I were obtained from the Japanese Collection of Research Bioresources (JCRB) Cell Bank. All cell lines except for NCI-H2009, NCI-H1048, SBC-5, and RMG-I were authenticated by sequencing and comparing short tandem repeats (STRs) (Promega Corporation) and tested negative for Mycoplasma using MycoAlert (Lonza, LT07-318). All cell lines were used for functional experiments within 2 months of passage post-receipt.

### Generation of lentiviruses and viral transduction

To generate lentiviral particles, 293LTV cells were transfected with lentiviral plasmids and packaging plasmids using Lipofectamine 3000 (Thermo Fisher Scientific; L3000015). After 16 h, the medium was replaced with fresh growth medium, and cells were incubated for an additional 48 h. Lentivirus-containing supernatants were harvested and concentrated by centrifugation using Lenti-X Concentrator (Takara; 631232). To establish the H23 isogenic model, SMARCA4-deficient H23 cells were transduced with lentiviral particles carrying human *SMARCA4* (pLV[Exp]-Puro-CMV>hSMARCA4[NM_001128849.1]; Vector Builder, VB181029-1161teb) in the presence of 8 μg/mL polybrene (Nacalai Tesque; 12996-81). After 24 h of incubation, the medium was replaced with fresh growth medium. Between 24 and 48 h post-transduction, cells were subjected to selection in growth medium containing 2 μg/mL puromycin (Wako; 160-23151) for 3–7 days. For PARD3B overexpression, cells were transduced with lentiviral particles encoding full-length *PARD3B* cDNA (Precision LentiORF PARD3B; Dharmacon, PLOHS_100061657, OHS5898-202623935). Cells were harvested 96 h post-transduction for immunoblotting or RNA extraction for RNA-seq analysis.

### siRNA screening, cell viability, and colony formation assays

For the chromatin remodeling factor screen, a custom siRNA library targeting 17 ATP-dependent chromatin remodeling factors (excluding SWI/SNF subunits) and NuRD complex components was synthesized by Dharmacon. To ensure robust gene silencing, a double transfection protocol was employed. Briefly, SWI/SNF-deficient H23 cells and SMARCA4-restored cells were seeded in 24-well plates at a density of 2 × 10^5^ cells per well and transfected with siRNAs (final concentration 20 nM) using Lipofectamine RNAiMAX (Thermo Fisher Scientific; 13778150) according to the manufacturer’s instructions. Forty-eight hours post-transfection, cells were trypsinized, reseeded in 24-well plates, and subjected to a second round of transfection with siRNAs (20 nM). After an additional 48 h, cells were trypsinized, counted, and re-plated into 96-well plates at a density of 500 cells per well. Cell viability was assessed 7 days after the final plating by measuring cellular ATP levels using the CellTiter-Glo Luminescent Cell Viability Assay (Promega; G7571). For colony formation assays, 1000 cells were seeded in six-well plates, treated as indicated, and cultured for 10–14 days. Colonies were fixed with methanol and stained with 0.5% crystal violet solution. The ON-TARGETplus SMARTpool siRNAs targeting *CHD3* (L-023015-00), *PARD3B* (L-017697-02), and Non-targeting control (D-001810-10) were purchased from Dharmacon. A complete list of siRNAs used in the screen is provided in Supplementary Table S3.

### Immunoblot analysis

To extract proteins, 5 × 10^5^ cells were harvested, washed with PBS, and lysed with 150 μL of 1× SDS sample buffer at 95 °C for 5 min. Chromatin was sonicated on ice (20 cycles of 15-second pulses; high setting; 15 s between pulses) using a Bioruptor (M&S Instruments). The cell lysates were quantified using Pierce 660 nm Protein Assay Reagent (Thermo Fisher Scientific, 22660) and Ionic Detergent Compatibility Reagent for Pierce™ 660 nm Protein Assay Reagent (Thermo Fisher Scientific, 22663). Next, 15 μg of protein was analyzed by immunoblotting. SDS–PAGE separated proteins, transferred them to PolyVinylidene Di-Fluoride (PVDF) membranes, and immunoblotted with the indicated antibodies. β-actin was used as a loading control. Membranes were blocked for 1 h at 25 °C with PVDF Blocking Reagent for Can Get Signal (TOYOBO, NYPBR01) and then probed for 1 hour at 25 °C with Can Get Signal Solution 1 (TOYOBO, NKB-201) containing primary antibodies. After washing with TBS containing 0.1% Tween 20, the membranes were incubated for 30 min at 25 °C with TBS containing 0.1% Tween 20, 1% BSA, and horseradish peroxidase-conjugated anti-mouse (CST, 7076) (RRID: AB_330924) or anti-rabbit (CST, 7074) (RRID: AB_2099233) secondary antibodies before visualization using Western Lightning ECL Pro (Perkin Elmer, NEL120001EA). Chemiluminescence signals were measured using a FUSION Chemiluminescence Imaging System (M&S Instruments). Antibodies specific to the proteins were used for immunoblotting, as provided in Supplementary Table S4.

### Densitometric analysis

For quantitative assessment of protein expression, the signal intensities of immunoblot bands were measured using ImageJ software (National Institutes of Health, Bethesda, MD, USA). The relative protein expression levels were determined by normalizing the target protein signal (e.g., PARD3B) to the corresponding loading control signal (β-Actin) from the same blot. The normalized ratio for the control sample was set to 1, and the relative fold-changes for experimental groups were calculated accordingly.

### GFP-based competition assay

To evaluate the long-term effect of CHD3 depletion on cell proliferation, we performed a GFP-based competition assay using the SMARTvector Inducible Lentiviral shRNA system (Horizon Discovery). SMARCA4/SMARCA2-deficient (H23) and SMARCA4/SMARCA2-proficient (H460) cells were transduced with lentiviral vectors encoding a Doxycycline (Dox)-inducible shRNA targeting *CHD3* (Catalog #V3IHSMCG_6422690) or a non-targeting control (Catalog #VSC11651). In this system, the expression of both the shRNA and the TurboGFP reporter is driven by the Tet-inducible promoter. Stable cell lines were established by Puromycin selection. For the competition assay, shRNA-transduced cells were mixed with parental isogenic cells at a 1:1 ratio. The mixed populations were cultured in the presence of Dox (1 µg/mL) to induce the expression of shRNA and TurboGFP. Cells were passaged every 2–3 days, at which time the culture medium containing Dox was refreshed to maintain induction. The percentage of TurboGFP-positive cells was monitored at the indicated time points using a Guava flow cytometer (Millipore). Relative cell viability was calculated by dividing the percentage of TurboGFP-positive cells at each indicated time point by the percentage of TurboGFP-positive cells at Day 0, and expressed as a percentage relative to the initial time point. A decrease in the relative viability indicates a proliferation defect or cell death induced by the shRNA.

### Quantitation of mRNA

To measure basal mRNA levels, 2 × 10^4^ cells were plated into 96-well plates and incubated for 24 h. For siRNA knockdown experiments, 2 × 10^4^ cells were plated into 96-well plates and transfected with siRNAs (20 nM) using Lipofectamine RNAiMAX (Thermo Fisher Scientific; 13778150). Cells were harvested after 48–96 h of incubation. For drug-treated cells, 2 × 10^4^ cells were plated into 96-well plates and incubated for 24 h. The medium was then replaced with a medium containing Vorinostat (Selleck, S1047) and incubated for 24 h. Cell lysis and cDNA synthesis were performed using the SuperPrep II Cell Lysis & RT Kit for qPCR (TOYOBO; SCQ-401) according to the manufacturer’s instructions. Quantitative PCR was performed using the THUNDERBIRD Probe qPCR Mix (TOYOBO; QPS101) and TaqMan Gene Expression Assays (Thermo Fisher Scientific). For the extraction of mRNA from tumor samples, the tumor xenograft samples were harvested, weighed, and washed with PBS. Tissue samples (~30 mg) were pulverized in liquid nitrogen and processed using the RNeasy Mini Kit (QIAGEN; 74104) in conjunction with QIAshredder columns (QIAGEN; 79654), according to the manufacturer’s protocol for animal tissues. RNA was eluted in a final volume of 50 μL. Subsequently, cDNA was synthesized using PrimeScript RT Master Mix (Perfect Real Time) (Takara Bio; RR036A). The following gene-specific primer/probe sets were used: *CHD3* (Hs01050212_m1), *PARD3B* (Hs01565182_m1), and *GAPDH* (Hs99999905_m1). PCR was performed on an ABI StepOnePlus Real-Time PCR System (Applied Biosystems; RRID:SCR_015805) under the following conditions: denaturation at 95 °C for 15 s, followed by annealing and extension at 60 °C for 30 s (40 cycles). Relative mRNA expression levels were calculated using the comparative Ct (2^−ΔΔCt^) method, with *GAPDH* serving as the internal control.

### RNA-seq analysis

Cells were transfected with siRNAs (20 nM) using Lipofectamine RNAiMAX (Thermo Fisher Scientific; 13778150) and harvested after 96 h of incubation. For overexpression experiments, cells were transduced with lentiviral cDNA expression vectors and harvested after 96 h. Total RNA was isolated using the RNeasy Mini Kit (Qiagen; 74104) according to the manufacturer’s instructions. Library preparation and sequencing were performed by Rhelixa, Inc. (Tokyo, Japan). mRNA was purified using the Poly(A) mRNA Magnetic Isolation Module (New England Biolabs), and libraries were constructed using the NEBNext Ultra II Directional RNA Library Prep Kit for Illumina (New England Biolabs). Sequencing was performed on an Illumina NovaSeq 6000 platform (150 bp paired-end reads).

### ATAC-seq analysis

Cells (2 × 10^5^) were cultured in 12-well plates and transfected with siRNAs (20 nM) using Lipofectamine RNAiMAX (Thermo Fisher Scientific; 13778150). After 96 h of incubation, cells were harvested. ATAC-seq library preparation and sequencing were performed by DNAFORM (Yokohama, Japan). Briefly, 1 × 10^5^ viable cells were lysed, and the transposition reaction was performed using Tn5 Transposase (Illumina; FC-121-1030) at 37 °C for 30 min. The transposed DNA was purified using the MinElute PCR Purification Kit (Qiagen; 28004). Library amplification was performed using NEBNext Q5 Hot Start HiFi PCR Master Mix (New England Biolabs; M0543S) and custom Nextera PCR primers^26^. Five cycles of initial PCR were conducted, and the number of additional cycles was determined by quantitative PCR (qPCR) analysis of the partially amplified products. The final libraries were purified using Agencourt AMPure XP beads (Beckman Coulter; A63881) with double size selection (ratios: 0.5× and 1.4×) according to the manufacturer’s protocol. Sequencing was performed on an MGI Tech DNBSEQ-G400R platform (150 bp paired-end reads). Data processing, including read mapping and peak calling, was performed using the ENCODE ATAC-seq pipeline (https://github.com/ENCODE-DCC/atac-seq-pipeline). Reads were mapped to the human reference genome (hg38) using Bowtie2 (v2.3.4.3)^27^, and duplicate reads were removed using Picard (v2.20.7) and samtools (v1.9). Peak calling was performed using MACS2 (v2.2.4) with default parameters. After the removal of blacklisted regions, peak consistency between replicates was assessed using the irreproducible discovery rate (IDR) framework (v2.0.4.2). Peak annotation was performed using HOMER (v4.9.1) with default settings. Differentially accessible peaks were identified using DESeq2 (v1.20.0).

### CUT&RUN-seq analysis

To assess proliferating cells, 5 × 10^5^ cells were seeded in six-well plates and incubated for 24 h. CUT&RUN analysis was performed using the CUT&RUN Assay Kit (Cell Signaling Technology [CST]; 86652). Cells were harvested by trypsinization, and 5 × 10^5^ cells were suspended in 1× Wash Buffer and bound to concanavalin A-coated beads. Cells were permeabilized with 100 μL of Antibody Binding Buffer containing digitonin and incubated at 4 °C for 2 h with 2 μL of the following primary antibodies: anti-H3K4me3 (CST, 9751; RRID:AB_2616028), anti-H3K4me1 (CST, 5326; RRID:AB_10695860), anti-H3K27ac (CST, 8173; RRID:AB_2616015), anti-H3K27me3 (CST, 9733; RRID:AB_2616029), anti-SMARCA4 (Abcam, ab110641; RRID:AB_10860265), and anti-CHD3 (Bethyl Laboratories, A301-219A; RRID:AB_890566). Cells were washed four times with Digitonin Buffer, incubated with pAG-MNase solution at 4 °C for 1 h, and washed an additional four times. MNase was activated by adding 3 μL of CaCl_2_ solution at 4 °C for 30 min, and the reaction was stopped by adding 1× Stop Buffer. For input DNA preparation, 1 × 10^5^ cells were treated with DNA extraction buffer containing RNase and Proteinase K, followed by incubation at 55 °C for 1 h. The genomic DNA was sonicated to yield fragments of 150–500 bp (40 cycles of 15 s on/30 s off, high setting) using a Bioruptor (M&S Instruments). Chromatin and input DNA were purified using DNA Purification Buffers and Spin Columns (CST; 14209). Libraries were constructed using the ThruPLEX DNA-Seq Kit (Takara; R400674) and amplified for 16 cycles (denaturation at 98 °C for 20 s; annealing and extension at 72 °C for 50 s). Sequencing was performed by Takara Bio using the Illumina NovaSeq 6000 platform (150 bp paired-end reads).

### Unified data processing pipeline

#### Data preprocessing and RNA-seq analysis

Raw sequencing reads from all applications were trimmed using fastp (version 0.12.4; RRID:SCR_016962)^28^. For RNA-seq data, trimmed reads were mapped to the human reference genome (hg38) using HISAT2 (version 2.2.1; RRID:SCR_015530)^29^. Gene expression levels were quantified as transcripts per million (TPM) using Strand NGS (version 4.0; TOMY Digital Biology). Differential expression analysis was performed using Strand NGS, identifying differentially expressed genes (DEGs) based on the criteria of *P* < 0.05 and |log2(Fold Change)| > 1. Venn diagram generation and WikiPathway analysis were subsequently performed on these DEG lists using Strand NGS (version 4.0).

#### ATAC-seq and CUT&RUN data processing

For ATAC-seq and CUT&RUN data, trimmed reads were mapped to hg38 using Bowtie2 (version 2.4.5; RRID:SCR_016368)^27^ with the following parameters: -k 1 --no-mixed --no-discordant -X 2000. PCR duplicates were removed using the MarkDuplicates command in Picard (version 2.26.11; RRID:SCR_006525) [http://broadinstitute.github.io/picard].

#### Visualization and genome browser tracks

Genome browser tracks (BigWig files) were generated using deepTools (version 3.5.1; RRID:SCR_016366)^30^. For ATAC-seq and RNA-seq data, the bamCoverage command was used with the following parameters: --normalizeUsing CPM --binSize 10 --smoothLength 30. For CUT&RUN data, background signals were subtracted using input controls. The bamCompare command was used with the following parameters: --operation subtract --normalizeUsing CPM --scaleFactorsMethod None --binSize 10 --smoothLength 30. The resulting BigWig files were visualized using the integrative genomics viewer (IGV) (version 2.13.2; RRID:SCR_011793)^31^.

#### Genomic signal distribution analysis

To visualize the genome-wide distribution of CUT&RUN signals relative to transcription start sites (TSS), we utilized the deepTools suite. Read coverage scores were calculated using the computeMatrix reference-point module. The analysis window was defined as 3 kb upstream and 3 kb downstream of the TSS, based on the GENCODE v38 annotation (hg38). Heatmaps were generated using the plotHeatmap module with *k*-means clustering (*k* = 3) to classify regions based on signal enrichment patterns.

### Gene set enrichment analysis (GSEA)

Read counts were generated using FeatureCounts (version 2.0.6)^32^. Gene set enrichment analysis (GSEA) was performed using the GSEA software (version 4.3.2)^33^ in conjunction with the Molecular Signatures Database (MSigDB)^33^. Specifically, the hallmark gene sets were utilized to identify significantly enriched biological pathways. Gene sets were considered statistically significant if they met the criteria of a false discovery rate (FDR) *q*-value < 0.25 and a nominal *P*-value < 0.05.

### Mouse xenograft model

The SBC-5 cell line was strategically selected for the in vivo xenograft studies. While multiple SMARCA4/SMARCA2 dual-deficient cell lines (e.g., H23, H1703) were validated in vitro, SBC-5 showed consistently superior tumor engraftment rates and growth kinetics in nude mice under our experimental conditions, making it the most robust and reproducible model for evaluating in vivo therapeutic efficacy. In the doxycycline (Dox) treatment study, cells were counted and resuspended in a 1:1 mixture of PBS and Matrigel (Corning; 354234) on ice. SBC-5 cells (5 × 10^5^ cells per mouse) were injected subcutaneously into the flank of 5–6-week-old female BALB/c-*nu/nu* mice (CLEA Japan). The total injection volume was 200 µL. When tumors became palpable (~14–21 days post-implantation), mice were randomized into two groups and fed either a diet containing Dox (625 ppm; Research Diets, D13110903) or a control diet (Research Diets, D10001). Tumor growth was monitored twice weekly using calipers. Tumor volume was calculated using the formula *V* = (*L* × *W*^2^)/2, where V is volume (mm^3^), *L* is the largest diameter (mm), and *W* is the smallest diameter (mm). At the experimental endpoint, mice were euthanized in accordance with institutional guidelines and standard protocols.

### Public dataset analysis

Clinical and transcriptomic data for the lung adenocarcinoma (LUAD) cohort from the TCGA PanCancer Atlas (*n* = 510) were accessed and downloaded via the cBioPortal for Cancer Genomics (https://www.cbioportal.org/). For the expression analysis of *PARD3B*, RNA-seq data—specifically batch-normalized RSEM (RNA-Seq by Expectation-Maximization) values derived from Illumina HiSeq_RNASeqV2—and the corresponding *SMARCA4* mutation statuses were extracted.

### Statistical analysis

Data were analyzed using GraphPad Prism software (version 10.4.1; GraphPad Software). Statistical significance was determined using the two-tailed Student’s *t*-test for continuous variables that approximated a normal distribution. For data that did not follow a normal distribution, such as the clinical RNA-seq expression data from the TCGA dataset, the two-sided Mann–Whitney *U* test was employed. Statistically significant differences are indicated by asterisks as follows: *p < 0.05, **p < 0.01, and ***p < 0.001.

## Supplementary information

Suppplementary Information

## Data Availability

All raw NGS data files (RNA-seq, ATAC-seq, and CUT&RUN-seq) and processed bigWig files have been deposited in the NCBI Gene Expression Omnibus (GEO) under the accession number GSE323137. The data that support the findings of this study are available in the manuscript and supplementary materials. Further inquiries can be directed to the corresponding authors.

## References

[1] Kadoch, C. et al. Proteomic and bioinformatic analysis of mammalian SWI/SNF complexes identifies extensive roles in human malignancy. *Nat. Genet.* **45**, 592–601 (2013). 23644491 10.1038/ng.2628 PMC3667980

[2] Witkowski, L. et al. Germline and somatic SMARCA4 mutations characterize small cell carcinoma of the ovary, hypercalcemic type. *Nat. Genet.* **46**, 438–443 (2014). 24658002 10.1038/ng.2931

[3] Le Loarer, F. et al. SMARCA4 inactivation defines a group of undifferentiated thoracic malignancies transcriptionally related to BAF-deficient sarcomas. *Nat. Genet.* **47**, 1200–1205 (2015). 26343384 10.1038/ng.3399

[4] Schoenfeld, A. J. et al. The genomic landscape of SMARCA4 alterations and associations with outcomes in patients with lung cancer. *Clin. Cancer Res.* **26**, 5701–5708 (2020). 32709715 10.1158/1078-0432.CCR-20-1825 PMC7641983

[5] Karnezis, A. N. et al. Dual loss of the SWI/SNF complex ATPases SMARCA4/BRG1 and SMARCA2/BRM is highly sensitive and specific for small cell carcinoma of the ovary, hypercalcaemic type. *J. Pathol.* **238**, 389–400 (2016). 26356327 10.1002/path.4633 PMC4832362

[6] Oike, T. et al. A synthetic lethality-based strategy to treat cancers harboring a genetic deficiency in the chromatin remodeling factor BRG1. *Cancer Res.* **73**, 5508–5518 (2013). 23872584 10.1158/0008-5472.CAN-12-4593

[7] Andrianteranagna, M. et al. SMARCA4-deficient rhabdoid tumours show intermediate molecular features between SMARCB1-deficient rhabdoid tumours and small cell carcinomas of the ovary, hypercalcaemic type. *J. Pathol.* **255**, 1–15 (2021). 33999421 10.1002/path.5705

[8] Marquez, S. B., Thompson, K. W., Lu, L. & Reisman, D. Beyond mutations: additional mechanisms and implications of SWI/SNF complex inactivation. *Front Oncol.* **4**, 372 (2014). 25774356 10.3389/fonc.2014.00372 PMC4343012

[9] Mittal, P. & Roberts, C. W. M. The SWI/SNF complex in cancer—biology, biomarkers and therapy. *Nat. Rev. Clin. Oncol.* **17**, 435–448 (2020). 32303701 10.1038/s41571-020-0357-3 PMC8723792

[10] Wilson, B. G. et al. Epigenetic antagonism between polycomb and SWI/SNF complexes during oncogenic transformation. *Cancer Cell* **18**, 316–328 (2010). 20951942 10.1016/j.ccr.2010.09.006 PMC2957473

[11] Bitler, B. G. et al. Synthetic lethality by targeting EZH2 methyltransferase activity in ARID1A-mutated cancers. *Nat. Med.* **21**, 231–238 (2015). 25686104 10.1038/nm.3799 PMC4352133

[12] Hoffman, G. R. et al. Functional epigenetics approach identifies BRM/SMARCA2 as a critical synthetic lethal target in BRG1-deficient cancers. *Proc. Natl. Acad. Sci. USA.* **111**, 3128–3133 (2014). 24520176 10.1073/pnas.1316793111 PMC3939885

[13] Wilson, B. G. et al. Residual complexes containing SMARCA2 (BRM) underlie the oncogenic drive of SMARCA4 (BRG1) mutation. *Mol. Cell. Biol.* **34**, 1136–1144 (2014). 24421395 10.1128/MCB.01372-13 PMC3958034

[14] Sasaki, M. et al. Targeting dependency on a paralog pair of CBP/p300 against de-repression of KREMEN2 in SMARCB1-deficient cancers. *Nat. Commun.* **15**, 4770 (2024). 38839769 10.1038/s41467-024-49063-w PMC11153594

[15] Sasaki, M., Kato, D., Yoshida, H., Shimizu, T. & Ogiwara, H. Efficacy of CBP/p300 dual inhibitors against derepression of KREMEN2 in cBAF-dDeficient cancers. *Cancer Res. Commun.* **5**, 24–38 (2025). 39625239 10.1158/2767-9764.CRC-24-0484 PMC11701801

[16] Clapier, C. R., Iwasa, J., Cairns, B. R. & Peterson, C. L. Mechanisms of action and regulation of ATP-dependent chromatin-remodelling complexes. *Nat. Rev. Mol. Cell Biol.* **18**, 407–422 (2017). 28512350 10.1038/nrm.2017.26 PMC8127953

[17] Tong, J. K., Hassig, C. A., Schnitzler, G. R., Kingston, R. E. & Schreiber, S. L. Chromatin deacetylation by an ATP-dependent nucleosome remodelling complex. *Nature* **395**, 917–921 (1998). 9804427 10.1038/27699

[18] Xue, Y. et al. NURD, a novel complex with both ATP-dependent chromatin-remodeling and histone deacetylase activities. *Mol. Cell* **2**, 851–861 (1998). 9885572 10.1016/s1097-2765(00)80299-3

[19] Bornelov, S. et al. The nucleosome remodeling and deacetylation complex modulates chromatin structure at sites of active transcription to fine-tune gene expression. *Mol. Cell* **71**, 56–72 e54 (2018). 30008319 10.1016/j.molcel.2018.06.003 PMC6039721

[20] Thompson, B. J. Par-3 family proteins in cell polarity & adhesion. *FEBS J.* **289**, 596–613 (2022). 33565714 10.1111/febs.15754 PMC9290619

[21] Januario, T. et al. PRC2-mediated repression of SMARCA2 predicts EZH2 inhibitor activity in SWI/SNF mutant tumors. *Proc. Natl. Acad. Sci. USA.* **114**, 12249–12254 (2017). 29087303 10.1073/pnas.1703966114 PMC5699030

[22] Hoffmeister, H. et al. CHD3 and CHD4 form distinct NuRD complexes with different yet overlapping functionality. *Nucleic Acids Res.* **45**, 10534–10554 (2017). 28977666 10.1093/nar/gkx711 PMC5737555

[23] Martin, B. J. E. et al. Global identification of SWI/SNF targets reveals compensation by EP400. *Cell* **186**, 5290–5307 e5226 (2023). 37922899 10.1016/j.cell.2023.10.006 PMC11307202

[24] Denslow, S. A. & Wade, P. A. The human Mi-2/NuRD complex and gene regulation. *Oncogene* **26**, 5433–5438 (2007). 17694084 10.1038/sj.onc.1210611

[25] Farnaby, W. et al. Publisher Correction: BAF complex vulnerabilities in cancer demonstrated via structure-based PROTAC design. *Nat. Chem. Biol.* **15**, 846 (2019). 31267096 10.1038/s41589-019-0329-z

[26] Buenrostro, J. D., Giresi, P. G., Zaba, L. C., Chang, H. Y. & Greenleaf, W. J. Transposition of native chromatin for fast and sensitive epigenomic profiling of open chromatin, DNA-binding proteins and nucleosome position. *Nat. Methods* **10**, 1213–1218 (2013). 24097267 10.1038/nmeth.2688 PMC3959825

[27] Langmead, B. & Salzberg, S. L. Fast gapped-read alignment with Bowtie 2. *Nat. Methods* **9**, 357–359 (2012). 22388286 10.1038/nmeth.1923 PMC3322381

[28] Chen, S., Zhou, Y., Chen, Y. & Gu, J. fastp: an ultra-fast all-in-one FASTQ preprocessor. *Bioinformatics* **34**, i884–i890 (2018). 30423086 10.1093/bioinformatics/bty560 PMC6129281

[29] Kim, D., Paggi, J. M., Park, C., Bennett, C. & Salzberg, S. L. Graph-based genome alignment and genotyping with HISAT2 and HISAT-genotype. *Nat. Biotechnol.* **37**, 907–915 (2019). 31375807 10.1038/s41587-019-0201-4 PMC7605509

[30] Ramirez, F., Dundar, F., Diehl, S., Gruning, B. A. & Manke, T. deepTools: a flexible platform for exploring deep-sequencing data. *Nucleic Acids Res.* **42**, W187–W191 (2014). 24799436 10.1093/nar/gku365 PMC4086134

[31] Robinson, J. T. et al. Integrative genomics viewer. *Nat. Biotechnol.* **29**, 24–26 (2011). 21221095 10.1038/nbt.1754 PMC3346182

[32] Liao, Y., Smyth, G. K. & Shi, W. featureCounts: an efficient general purpose program for assigning sequence reads to genomic features. *Bioinformatics* **30**, 923–930 (2014). 24227677 10.1093/bioinformatics/btt656

[33] Subramanian, A. et al. Gene set enrichment analysis: a knowledge-based approach for interpreting genome-wide expression profiles. *Proc. Natl. Acad. Sci. USA.* **102**, 15545–15550 (2005). 16199517 10.1073/pnas.0506580102 PMC1239896

