# Supplementary material for: Targeting the CHD3 chromatin remodeler exploits a synthetic lethal vulnerability in dual SMARCA4/SMARCA2-deficient cancers via derepression of PARD3B
Source: NPJ Precis Oncol. 2026 May 22;10:315. doi: 10.1038/s41698-026-01499-7 (PMC13469703; doi:10.1038/s41698-026-01499-7)
Supplement: Supplementary file 1 [file 41698_2026_1499_MOESM1_ESM.pdf]

Supplementary Information

Title

Targeting the CHD3 Chromatin Remodeler Exploits a Synthetic Lethal Vulnerability in Dual SMARCA4/SMARCA2-Deficient Cancers via Derepression of PARD3B

Authors

Mariko Takeuchi, Yoshie Okimoto, Makoto Fukushima, Harumi Hirano, Hideaki Ogiwara*

Affiliations

Division of Cancer Therapeutics, National Cancer Center Research Institute,
Tsukiji 5-1-1, Chuo-ku, Tokyo, 104-0045, Japan

Supplementary Figure 1

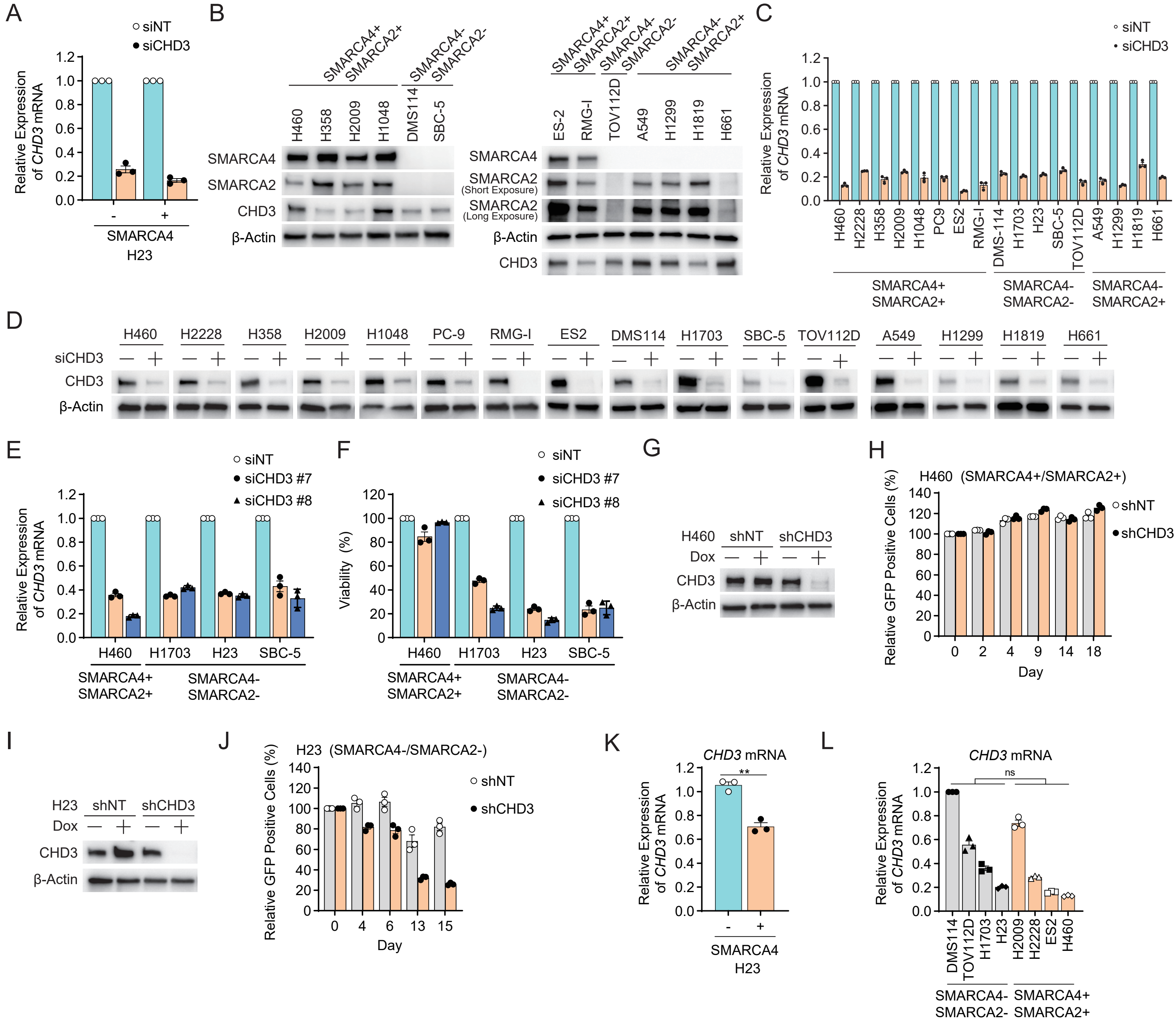

Supplementary Figure S1. Targeted Screening Identifies CHD3 as a Prominent Synthetic Lethal Vulnerability in Dual SMARCA4/SMARCA2-Deficient Cancers, Related to Figure 1

(A) RT-qPCR analysis of *CHD3* mRNA expression in the H23 isogenic model (SMARCA4-deficient vs. SMARCA4-restored) treated with control (siNT) or *CHD3*-targeting siRNA (siCHD3) for 96 h. Data are mean ± SD (n=3).

(B) Immunoblot analysis showing the expression levels of SMARCA4, SMARCA2, and CHD3 across the panel of cell lines used in this study. β-Actin served as a loading control.

(C, D) *CHD3* mRNA expression (C) and CHD3 protein expression (D) of SMARCA4/SMARCA2-proficient and single-deficient cell lines and dual SMARCA4/SMARCA2-deficient cell lines treated with siNT or siCHD3 for 96 h. β-Actin served as a loading control. For (C), data are mean ± SD (n=3)

(E, F) Validation of CHD3 dependency using two distinct siRNA sequences (siCHD3 #7 and #8). (E) RT-qPCR analysis of CHD3 mRNA expression in SMARCA4/SMARCA2-proficient H460 cells and three dual SMARCA4/SMARCA2-deficient cell lines (H1703, H23, SBC-5) treated with siNT or siCHD3 (siCHD3 #7 and #8) for 96 h, and (F) viability was assessed 7 days post-transfection. Data are mean ± SD (n=3).

(G–J) Evaluation of long-term CHD3 dependency using a doxycycline (Dox)-inducible shRNA system. SMARCA4/SMARCA2-proficient H460 cells (G, H) and dual SMARCA4/SMARCA2-deficient H23 cells (I, J) expressing GFP-tagged shNT or shCHD3 were cultured with or without Dox. Immunoblot analysis showing the expression levels of SMARCA4, SMARCA2, and CHD3 in SMARCA4/SMARCA2-proficient H460 cells (G) and dual SMARCA4/SMARCA2-deficient H23 cells (I). β-Actin served as a loading control. The ratio of GFP-positive cells was monitored by flow cytometry. Relative cell viability was calculated based on the depletion of GFP-positive (shRNA-expressing) cells relative to GFP-negative cells in a competition assay (H, J). Data are mean ± SD (n=3).

(K) RT-qPCR analysis showing the downregulation of *CHD3* mRNA upon SMARCA4 restoration in H23 cells. Data are mean ± SD (n=3).

(L) Basal *CHD3* mRNA expression levels across the cell line panel, grouped by SMARCA4/SMARCA2 deficiency status. The panel includes dual SMARCA4/SMARCA2-deficient cell lines (n=4) and SMARCA4/SMARCA2-proficient cell lines (n=4). Data are presented as mean ± SD of three replicates per cell line. ns, not significant (Student's t-test comparing the two groups).

*P < 0.05, **P < 0.01, ***P < 0.001 (Student's t-test).

Supplementary Figure 2

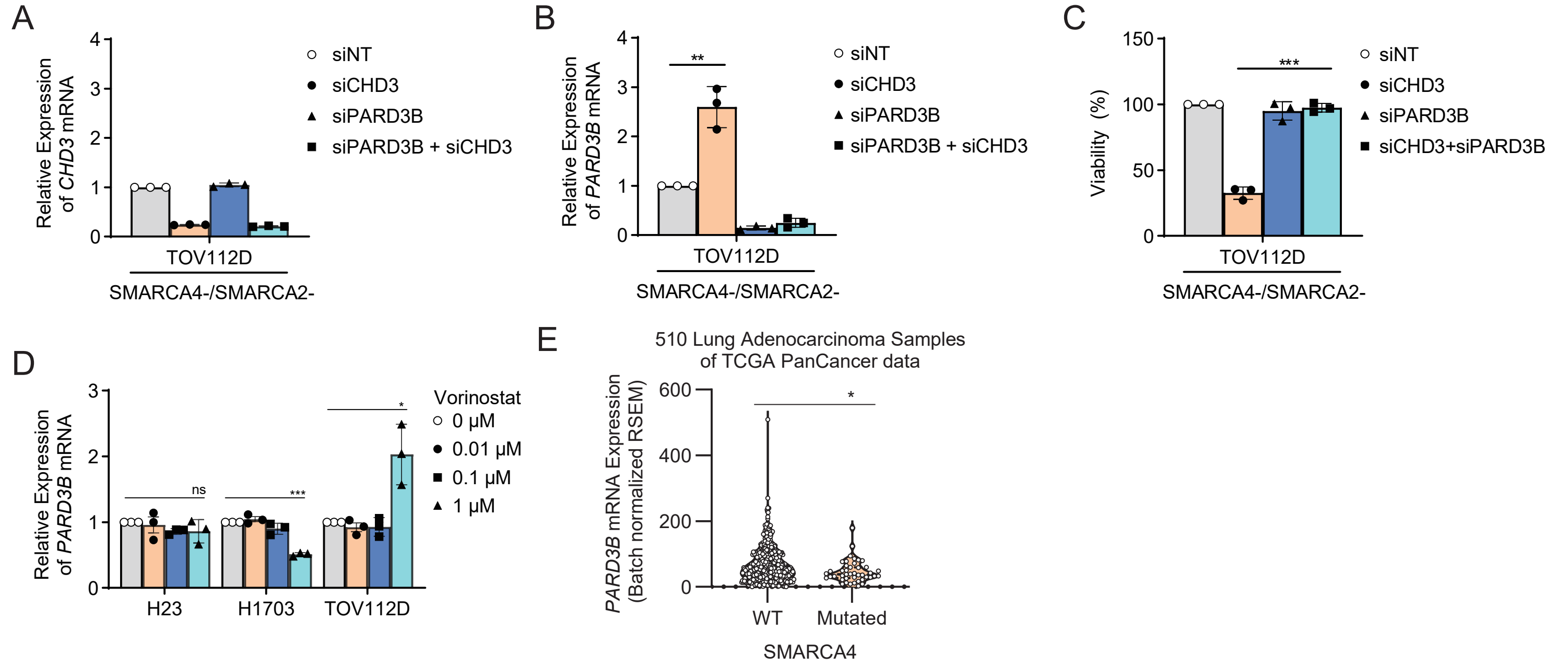

Supplementary Figure S2. Derepression of PARD3B Drives CHD3 Synthetic Lethality, Related to Figure 2
(A, B) RT-qPCR analysis of *CHD3* (A) and *PARD3B* (B) mRNA levels in the SWI/SNF-deficient ovarian cancer cell line TOV112D treated with siNT, siCHD3, siPARD3B, or the combination for 96 h. Data are mean ± SD (n=3).
(C) Cell viability of TOV112D cells treated as indicated for 7 days. Consistent with lung cancer models, concomitant depletion of PARD3B significantly rescues the cell viability defect caused by *CHD3* knockdown. Data are mean ± SD (n=3).
(D) Effect of HDAC inhibition on *PARD3B* expression. SWI/SNF-deficient cell lines (H23, H1703, and TOV112D) were treated with the indicated concentration of the HDAC inhibitor Vorinostat or vehicle control (DMSO) for 24 h. *PARD3B* expression levels were analyzed. Note the heterogeneous response across cell lines, indicating that HDAC inhibition does not consistently recapitulate the phenotype of CHD3 loss. Data are mean ± SD (n=3).
(E) Clinical validation of *PARD3B* suppression in *SMARCA4*-deficient tumors. Violin plot showing the basal mRNA expression levels (batch normalized from Illumina HiSeq_RNASeqV2, RSEM) of *PARD3B* in Lung Adenocarcinoma samples from the TCGA PanCancer Atlas dataset (n = 510). Samples are stratified by SMARCA4 mutation status (Wild-Type [WT] (n = 467) vs. Mutated (n = 43)). The solid line within the violin represents the median, and the dashed lines represent the quartiles. Statistical significance was determined using the two-sided Mann-Whitney U test (*P < 0.05), which accounts for the non-normal distribution of the RNA-seq expression data.
*P < 0.05, **P < 0.01, ***P < 0.001 (Student's t-test, , unless otherwise indicated).

Supplementary Figure 3

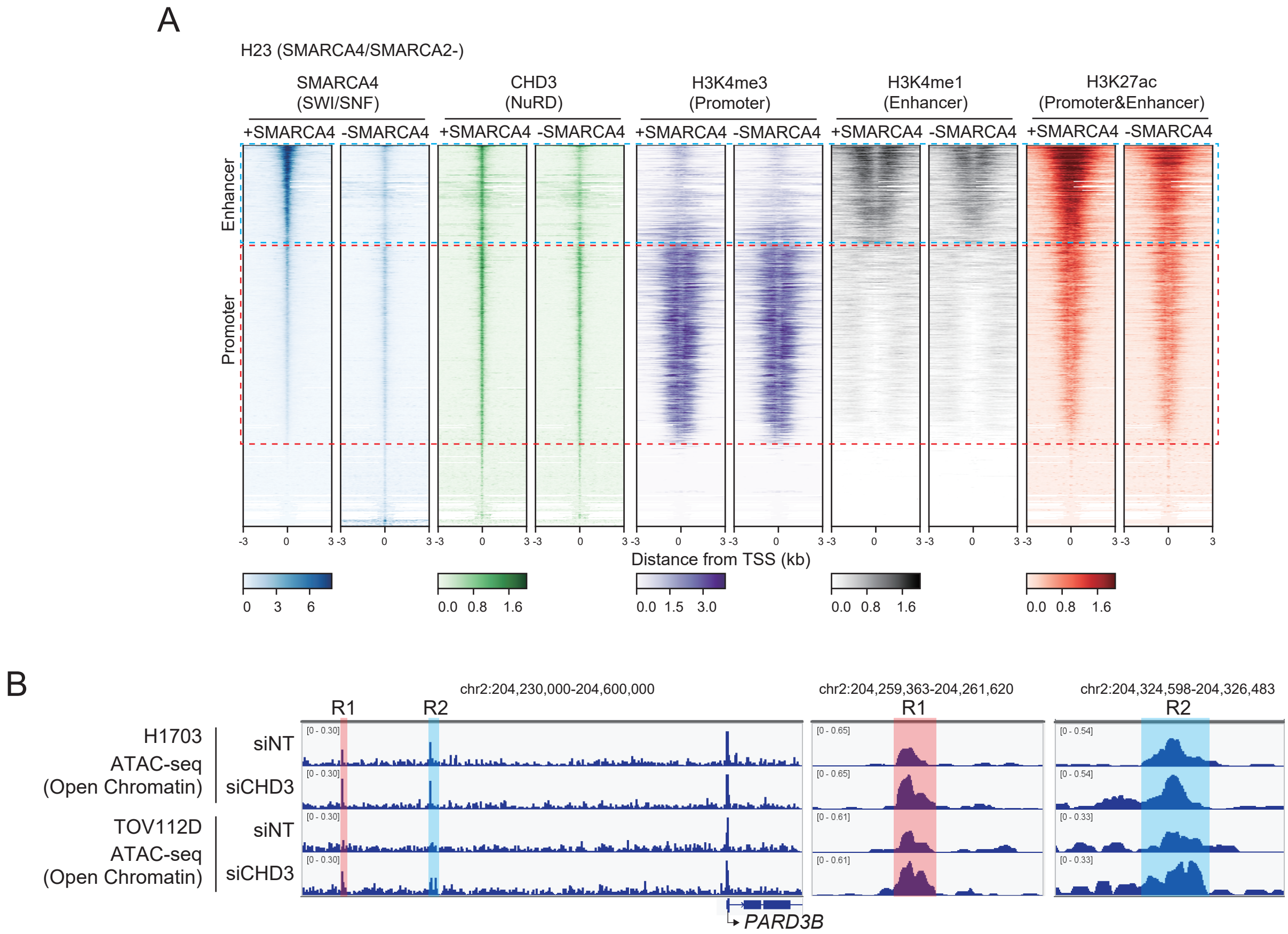

Supplementary Figure S3. CHD3 Maintains Chromatin Compaction at the PARD3B Enhancer in Dual SMARCA4/SMARCA2-Deficient Cells, Related to Figure 3

(A) Heatmaps centered on SMARCA4 peaks showing the genome-wide distribution of SMARCA4, CHD3, and histone modifications (H3K4me3, H3K4me1, H3K27ac) in H23 isogenic cells. Regions are classified into promoter and enhancer elements.

(B) Genome browser tracks of ATAC-seq signals at the PARD3B locus in daul SMARCA4/SMARCA2-deficient lung (H1703) and ovarian (TOV112D) cancer cell lines treated with siNT or siCHD3 for 96 h. Similar to H23 cells, CHD3 depletion induces increased chromatin accessibility at the 5' regulatory regions (R1, corresponding to E1) of the *PARD3B* gene.

Supplementary Figure 4

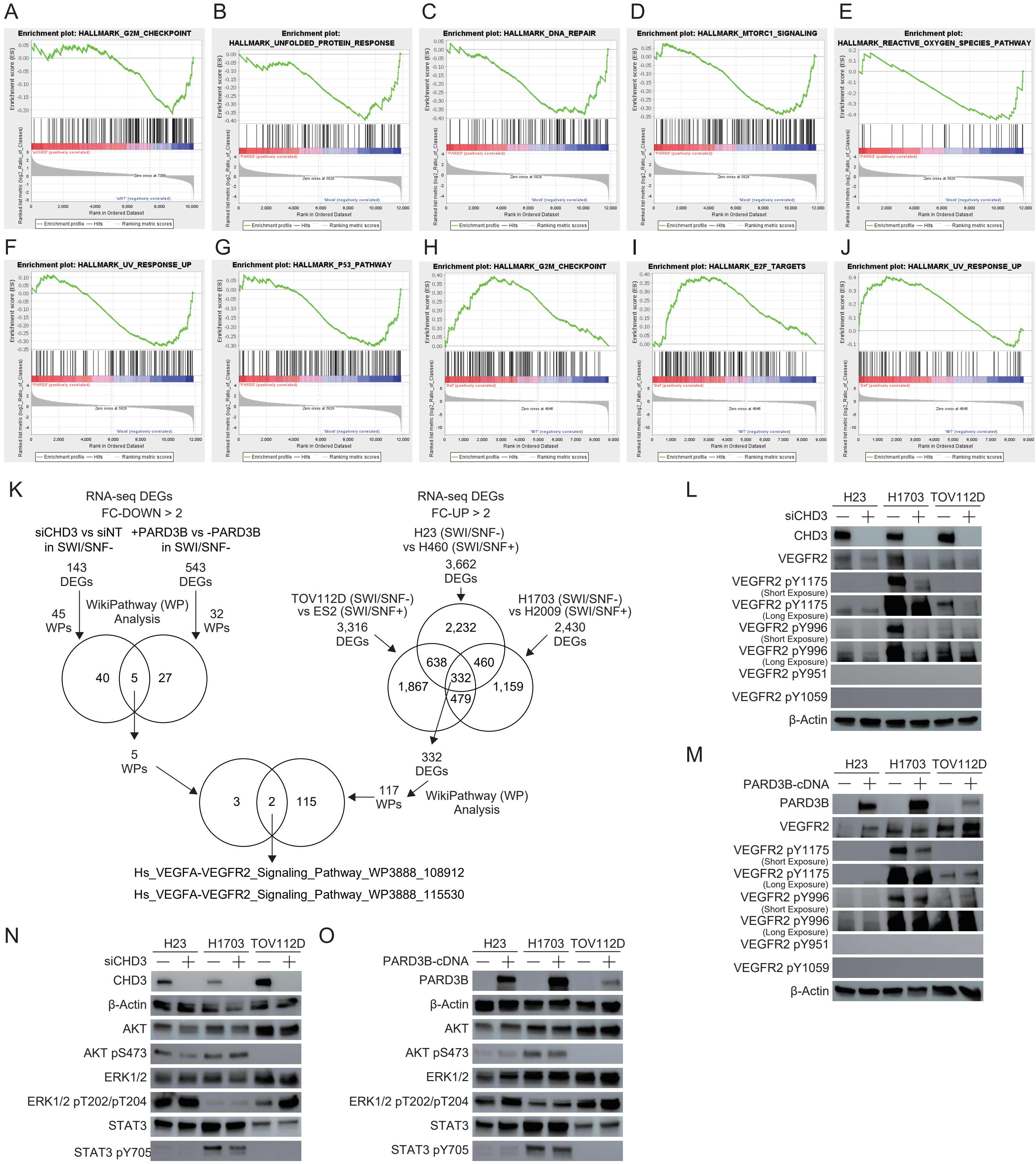

Supplementary Figure S4. PARD3B Accumulation is Associated with the Attenuation of MYC Signaling Signatures, Related to Figure 4

(A–G) Additional GSEA plots. (A) Suppression of *G2M_CHECKPOINT* in siCHD3-treated H23 cells. (B–E) Suppression of *UNFOLDED_PROTEIN_RESPONSE* (B), *DNA_REPAIR* (C), *MTORC1_SIGNALING* (D), and *REACTIVE_OXYGEN_SPECIES_PATHWAY* (E) in PARD3B-overexpressing H23 cells. (F, G) Enrichment of *UV_RESPONSE_UP* (F) and *P53_PATHWAY* (G) in PARD3B-overexpressing cells.
(H–J) Additional GSEA plots comparing dual SMARCA4/SMARCA2-deficient vs. SMARCA4/SMARCA2-proficient cells. Enrichment of *G2M_CHECKPOINT* (H), *E2F_TARGETS* (I), and *UV_RESPONSE_UP* (J) in dual SMARCA4/SMARCA2-deficient cells.
(K) WikiPathway analysis identifying the "*VEGFA-VEGFR2 Signaling Pathway*" as a common target downregulated by CHD3 suppression/PARD3B OE (left venn) and upregulated in dual SMARCA4/SMARCA2-deficient cells (right venn).
(L, M) Immunoblot analysis of VEGFR2 and its phosphorylation sites (pY1175, pY996, pY951, pY1059) in dual SMARCA4/SMARCA2-deficient cell lines (H23, H1703, TOV112D) treated for 96 h with siNT vs. siCHD3 (L) or Mock vs. *PARD3B*-cDNA (M).
(N, O) Immunoblot analysis of downstream signaling effectors (AKT, p-AKT, ERK1/2, p-ERK1/2, STAT3, p-STAT3) in dual SMARCA4/SMARCA2-deficient cell lines treated for 96 h with siNT vs. siCHD3 (N) or Mock vs. *PARD3B*-cDNA (O). Note the heterogeneous attenuation patterns across cell lines, suggesting context-dependent perturbation rather than uniform blockade. Statistical significance for GSEA was defined as Nominal p-value < 0.05 and FDR q-value < 0.25. Immunoblots are representative of at least two independent experiments.

Supplementary Table S1

Cell Lines	*SMARCA4* Gene Status	*SMARCA2* Gene Status	Protein Expression Status (Classification)
H460	*E672D*	WT	SMARCA4+/SMARCA2+
H2228	WT	WT	SMARCA4+/SMARCA2+
H358	WT	WT	SMARCA4+/SMARCA2+
H2009	WT	WT	SMARCA4+/SMARCA2+
H1048	WT	WT	SMARCA4+/SMARCA2+
PC9	WT	WT	SMARCA4+/SMARCA2+
ES2	WT	WT	SMARCA4+/SMARCA2+
RMG-I	WT	WT	SMARCA4+/SMARCA2+
DMS114	*E1310**	WT	SMARCA4-/SMARCA2-
H1703	*X668_splice*	WT	SMARCA4-/SMARCA2-
H23	*K1598_E1599delinsN**	WT	SMARCA4-/SMARCA2-
SBC-5	*A1245Lfs*13*	*W892L*	SMARCA4-/SMARCA2-
TOV112D	*L639Wfs*7*	WT	SMARCA4-/SMARCA2-
A549	*Q729Cfs*4*	WT	SMARCA4-/SMARCA2+
H1299	*X578_splice*	WT	SMARCA4-/SMARCA2+
H1819	*L1085fs*32*	WT	SMARCA4-/SMARCA2+
H661	*L1161Sfs*3/E672D*	WT	SMARCA4-/SMARCA2+

Supplementary Table S1. Genetic alteration and protein expression status of SMARCA4 and SMARCA2 in the evaluated cell line panel.
Mutation data were retrieved from the Cancer Cell Line Encyclopedia (CCLE, Broad Institute). In this study, the functional classification of SWI/SNF deficiency (Dual SMARCA4/SMARCA2-deficient, Single SMARCA4-deficient, or SMAMRCA4/SMARCA2-proficient) is defined strictly by endogenous protein expression levels confirmed via immunoblotting, rather than gene mutation status alone. Notably, while SMARCA4 loss is primarily driven by truncating or frameshift mutations, the concurrent loss of SMARCA2 protein in dual-deficient lines (e.g., H23, H1703) frequently occurs despite a wild-type *SMARCA2* gene, a phenomenon known to be driven by epigenetic silencing. Note: The H460 cell line harbors an E672D missense mutation in *SMARCA4*; however, this variant is of unknown oncogenic effect (allele frequency: 0.40). Because H460 cells robustly express both SMARCA4 and SMARCA2 proteins and are functionally insensitive to CHD3 depletion, they are classified as SWI/SNF-proficient (SMARCA4+/SMARCA2+) in our analyses.

Supplementary Table S2

Gene Symbol	RNA-seq Log2 FC (siCHD3/siNT)	ATAC-seq Log2 FC (siCHD3/siNT)	Merged Peak Region (hg38)	Distance to TSS (bp)
SERTAD4-AS1	2.73	2.25	chr1_210239887_210240133	-6,125
GLRB	1.71	1.01	chr4_157043029_157043539	-32,841
PARD3B	1.64	1.03	chr2_204339942_204340561	-205,223
PMS2P7	1.48	-1.09	chr7_75358603_75359598	-63
BCYRN1	1.44	1.59	chr2_47319756_47320302	-15,286
LMNTD1	1.38	1.54	chr12_25730720_25731489	-82,527
LOC100652999	1.34	1.07	chr12_53561595_53562049	47,838
SPATA18	1.34	2.24	chr4_52103416_52103643	52,226
RNVU1-1	1.30	1.07	chr1_148353376_148354034	8,828
HDAC9	1.28	1.47	chr7_18215507_18215756	-74,460
MAPRE3	1.27	1.22	chr2_26982448_26982820	11,997
DTNA	1.26	1.54	chr18_34738314_34738850	-17,394
C2orf76	1.23	1.03	chr2_119310760_119311590	55,567
GEM	1.21	-1.00	chr8_94259094_94259492	3,026
CEP112	1.17	1.24	chr17_65987676_65988340	-161,465
ZC3H12C	1.15	1.21	chr11_109939225_109939695	-153,932
CHGB	1.06	1.03	chr20_5918938_5919547	7,733
LOC100288798	1.06	-1.16	chr12_46392546_46393121	8,727
PTPRM	1.01	1.70	chr18_7877532_7878017	310,459
LOC105377590	-1.05	1.06	chr4_185470839_185471070	-476
ASGR1	-1.08	1.12	chr17_7191491_7192072	-12,218
MIR4519	-1.20	1.42	chr16_30839152_30839365	36,064
SPRY4-AS1	-1.26	-1.04	chr5_142472206_142472716	147,168
ACP7	-1.41	-1.39	chr19_39078587_39079410	-5,369
WT1-AS	-1.55	1.11	chr11_32487157_32487640	51,360
LINC01117	-1.76	1.01	chr2_176830262_176830856	192,805
PLAU	-1.84	-1.05	chr10_73908889_73909781	-1,766
TGFBI	-1.94	-1.63	chr5_136053588_136053944	24,778

Supplementary Table S2. List of 28 candidate genes showing altered expression and chromatin accessibility upon CHD3 knockdown in dual SMARCA4/SMARCA2-deficient H23 cells.
This table lists the 28 candidate genes identified from the intersection of RNA-seq and ATAC-seq analyses in the H23 isogenic model (as shown in Figure 2A, left panel). Values represent the log2 fold-change (FC) of mRNA expression and ATAC-seq peak accessibility in CHD3-depleted (siCHD3) cells compared to control (siNT) cells. The genomic coordinates of the altered ATAC-seq peaks and their distance to the transcription start site (TSS) are also provided. Negative values in Log2 FC indicate downregulation or decreased accessibility.

Supplementary Table S3

Gene Symbol	Catalog Number
CHD3	L-023015-00
SMARCA1	L-011392-00
SMARCA5	L-011478-00
INO80	L-004176-01
CHD4	L-009774-00
EP400	L-021272-02
CHD5	L-009878-00
SRCAP	L-004830-00
CHD1L	L-014368-00
CHD6	L-017349-00
CHD7	L-025947-01
CHD1	L-008529-00
CHD2	L-008948-03
CHD8	L-030271-01
SMARCAD1	L-013801-00
HELLS	L-017444-01
CHD9	L-031153-00
GATAD2A	L-015311-01
GATAD2B	L-013892-00
HDAC1	L-003493-00
HDAC2	L-003495-02
MBD2	L-011555-00
MBD3	L-013616-00
MTA1	L-004127-00
MTA2	L-008482-00
MTA3	L-022665-01
RBBP4	L-012137-00
RBBP7	L-011375-00
CDK2AP1	L-011902-00
PARD3B	L-017697-02
CHD3 (#7)	J-023015-07
CHD3 (#8)	J-023015-08

Supplementary Table S3. List of siRNAs used in this study.
Dharmacon ON-TARGETplus siRNAs were utilized for RNA interference experiments. In the provided catalog numbers, the prefix "L-" designates SMARTpool reagents. Each SMARTpool represents a pre-designed mixture of four distinct siRNA sequences targeting different regions of the specified gene transcript. This pooling strategy enables robust target knockdown at lower individual siRNA concentrations, thereby minimizing non-specific off-target effects. (Note: Catalog numbers beginning with the prefix "J-" indicate individual, single-sequence siRNAs used for validation). Specific catalog numbers are provided to ensure exact reproducibility without exhaustively listing all individual proprietary sequences.

Supplementary Table S4

Antibody	Catalog. Number	Supplier	RRID
SMARCA4	49360	Cell Signaling Technology	RRID:AB_2728743
β-Actin	3700	Cell Signaling Technology	RRID:AB_2242334
CHD3	ab109195	Abcam	RRID:AB_10862514
SMARCA2	11966	Cell Signaling Technology	RRID:AB_2797783
PARD3B	sc-398761	SantaCruz Biotechnology	-
MYC	5605P	Cell Signaling Technology	RRID:AB_1903938
VEGFR2	9698	Cell Signaling Technology	RRID:AB_11178792
VEGFR2 pTyr951	4991	Cell Signaling Technology	RRID:AB_331398
VEGFR2 pTyr996	2474	Cell Signaling Technology	RRID:AB_331023
VEGFR2 pTyr1059	3817	Cell Signaling Technology	RRID:AB_2132351
VEGFR2 pTyr1175	3770	Cell Signaling Technology	RRID:AB_1642326
AKT	2920	Cell Signaling Technology	RRID:AB_1147620
AKT pSer473	4060	Cell Signaling Technology	RRID:AB_2315049
ERK1/2	4695	Cell Signaling Technology	RRID:AB_390779
ERK1/2 pT202/pT204	4370	Cell Signaling Technology	RRID:AB_2315112
STAT3	9139	Cell Signaling Technology	RRID:AB_331757
STAT3 pT705	9145	Cell Signaling Technology	RRID:AB_2491009

Supplementary Table S4. Antibodies used in this study for Immunoblotting.

Full Uncropped Gels and Blots (Original Data)

Description: This supplemental file provides the full, uncropped images for all immunoblotting data presented in the manuscript.
Annotations: All target protein bands are enclosed in red boxes, with their corresponding molecular weights (kDa) indicated to confirm the identity of the bands.

Presentation Formats:
Original data are presented in one of the following formats:
A full-membrane chemiluminescence image displayed alongside its corresponding white-light photograph showing the molecular weight markers.
A merged image where the chemiluminescence signal is overlaid with the white-light molecular weight marker signals.

Note on Molecular Weight Markers: For specific datasets (Figures 1A, 1C, 1E, 5C, 5G, and Supplementary Figures 1G and 1I), white-light marker photographs were not recorded. However, the identity of these bands was strictly confirmed based on the use of identical antibodies and validated electrophoresis protocols consistent with other biological replicates in this study where molecular weight markers were fully documented. The consistency of migration patterns across multiple independent experiments ensures that all indicated bands accurately represent the specified target proteins.

Figure 1

A

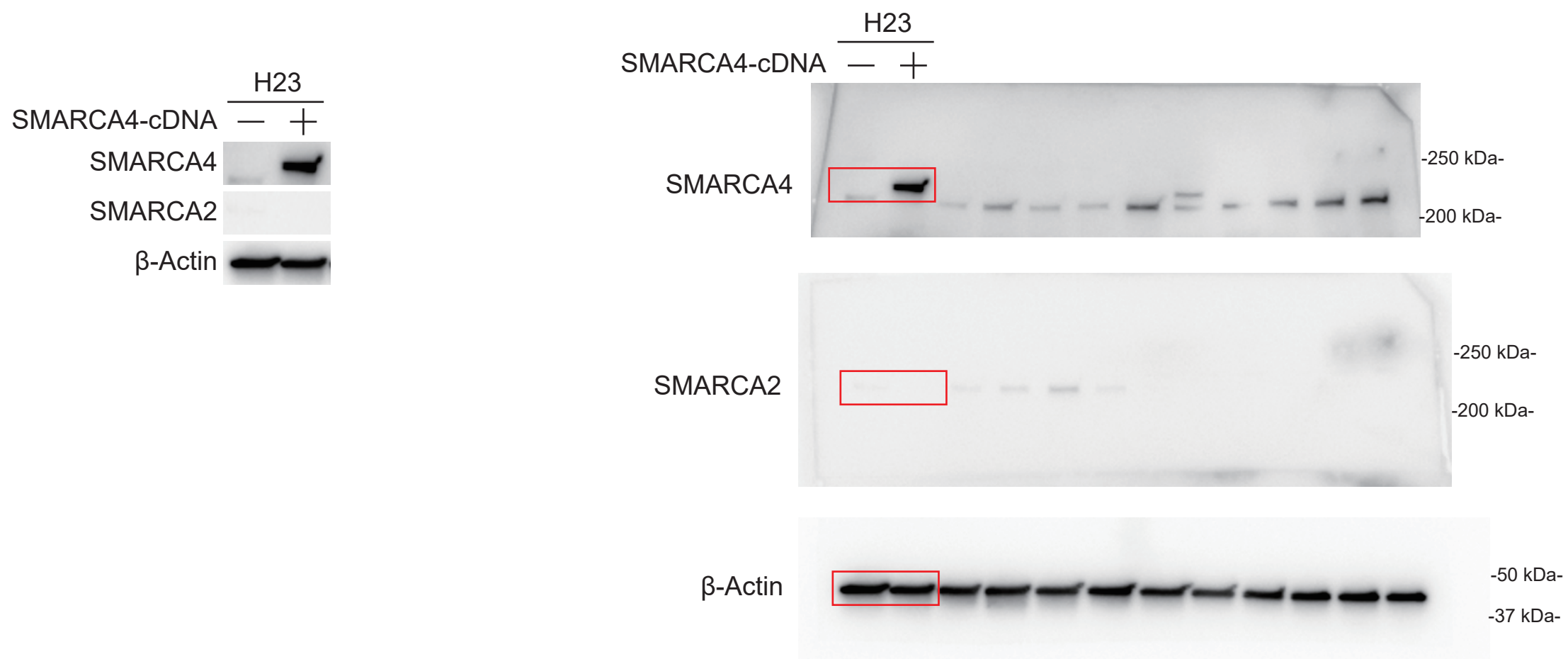

C

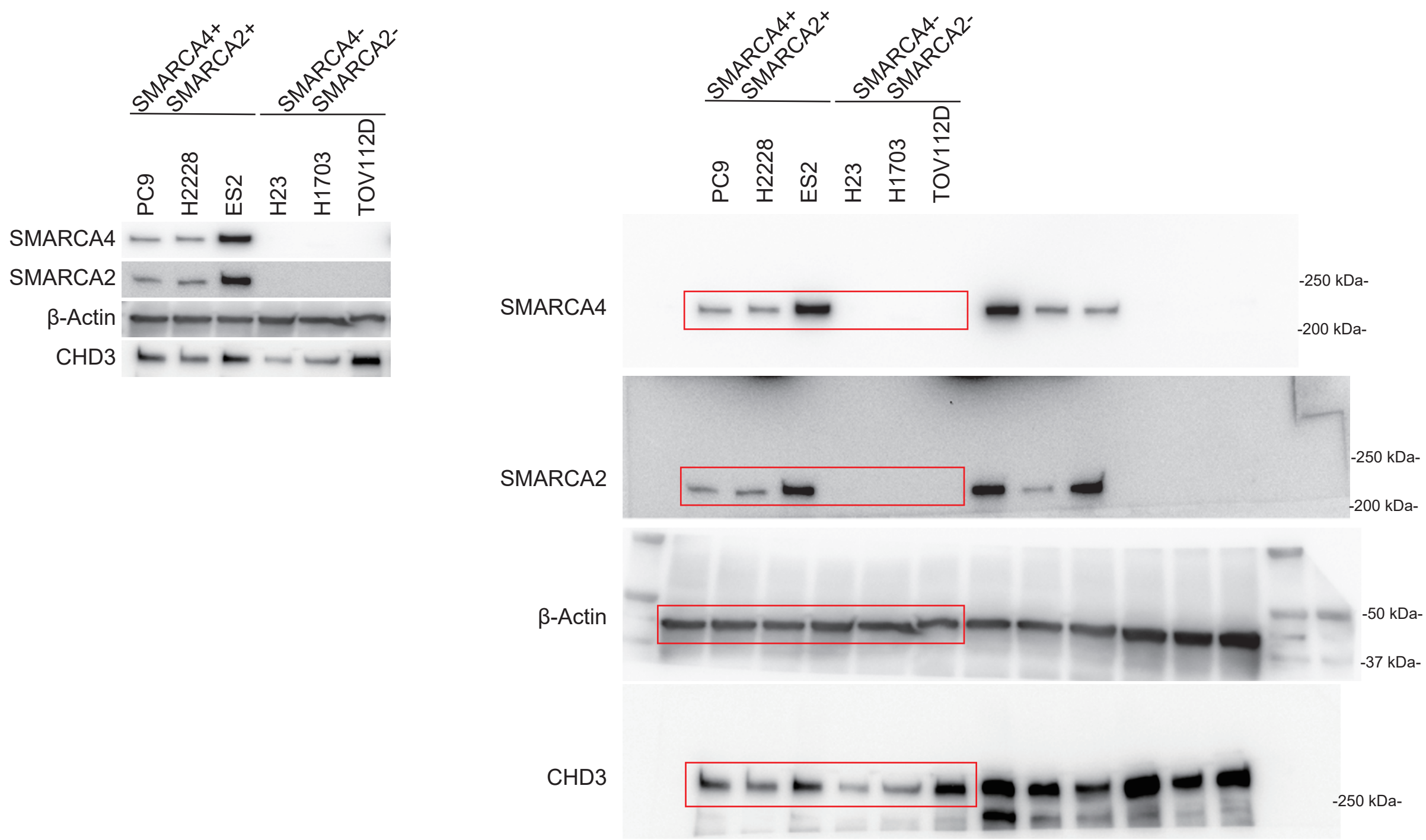

E

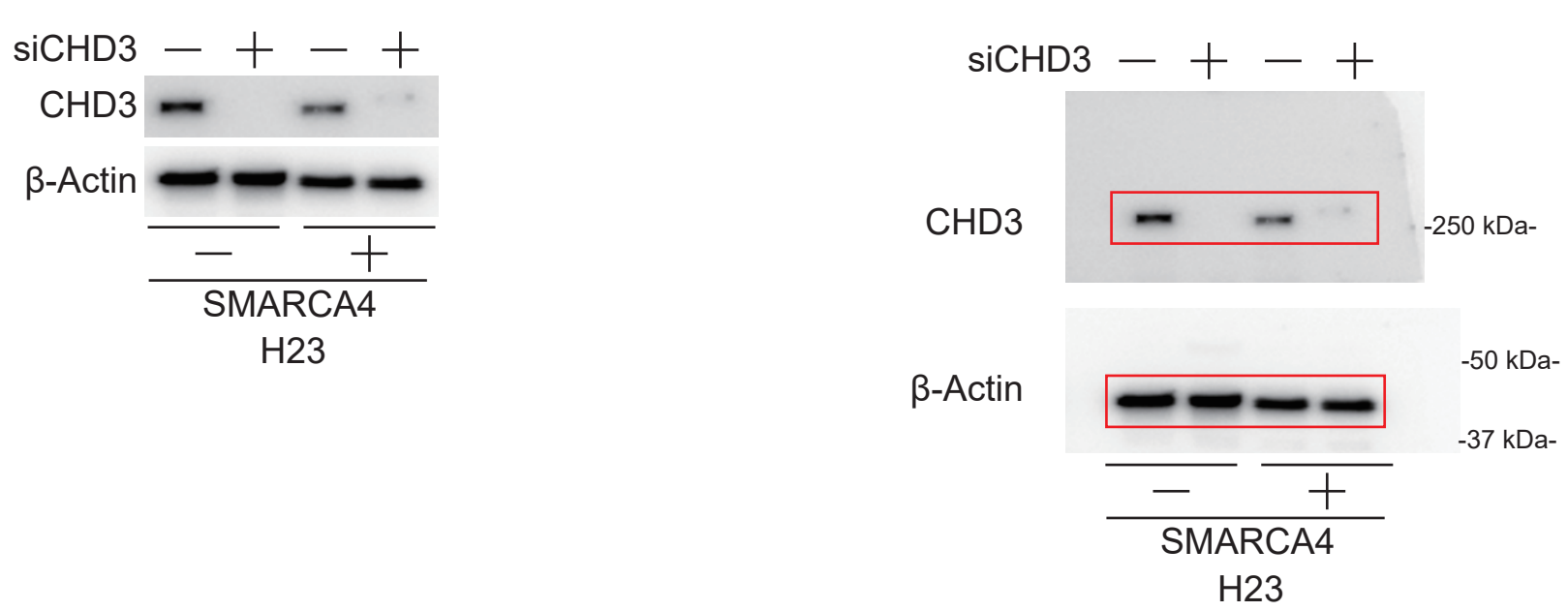

Supplementary Figure 1

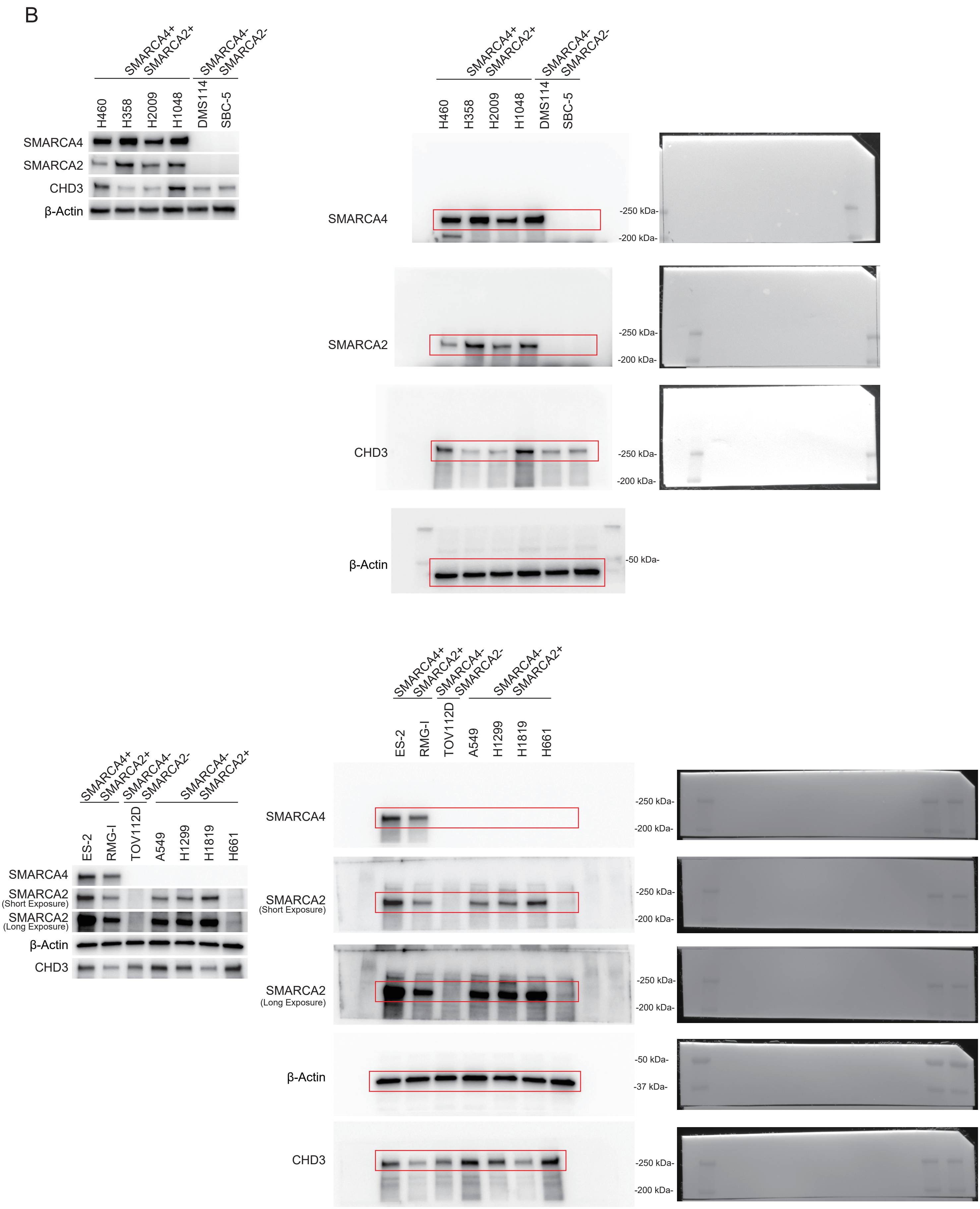

Supplementary Figure 1

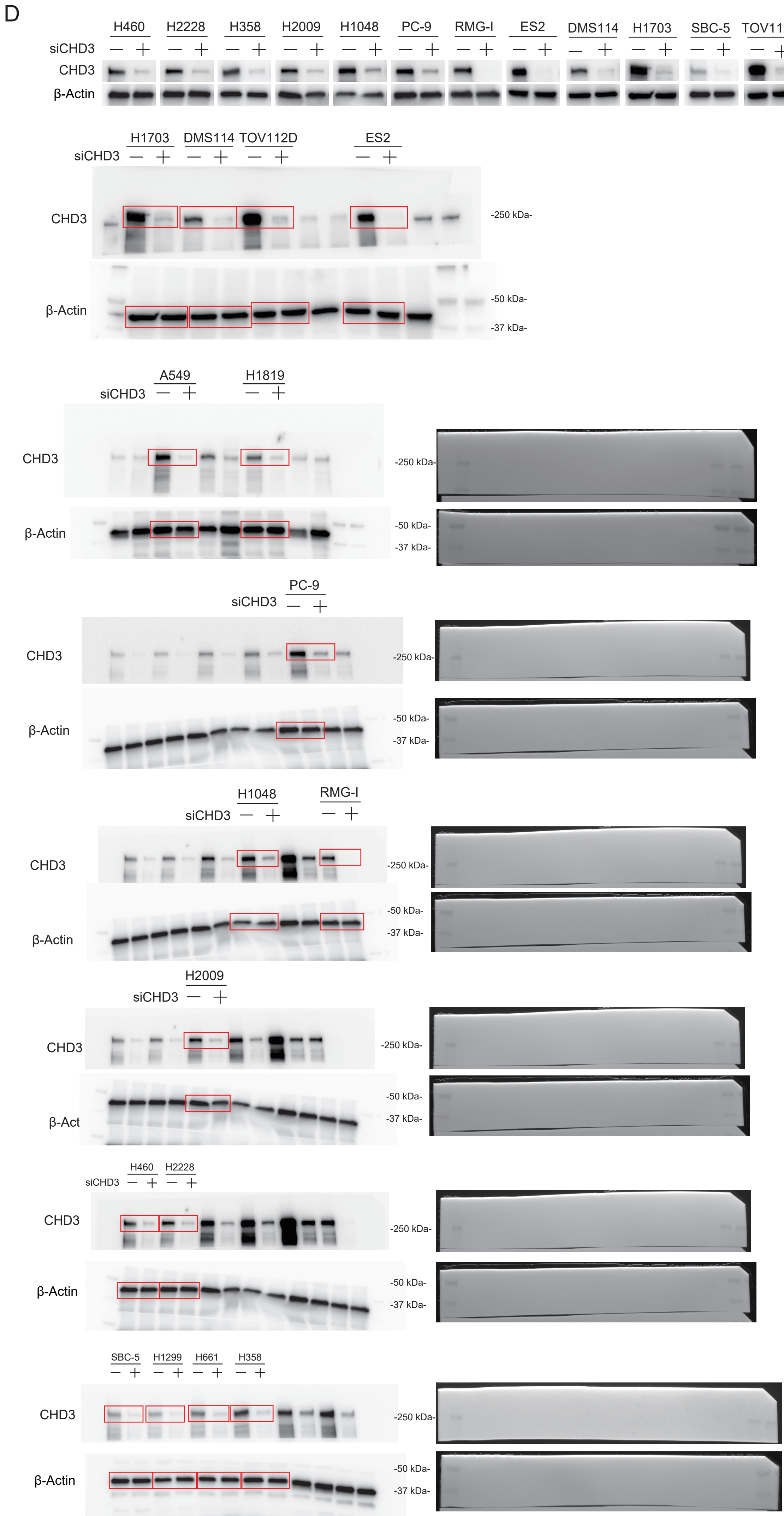

Supplementary Figure 1

G

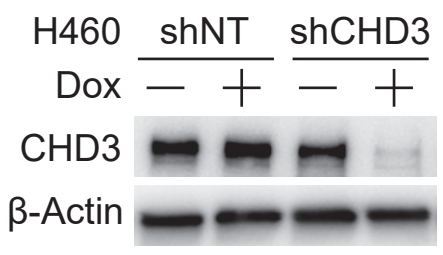

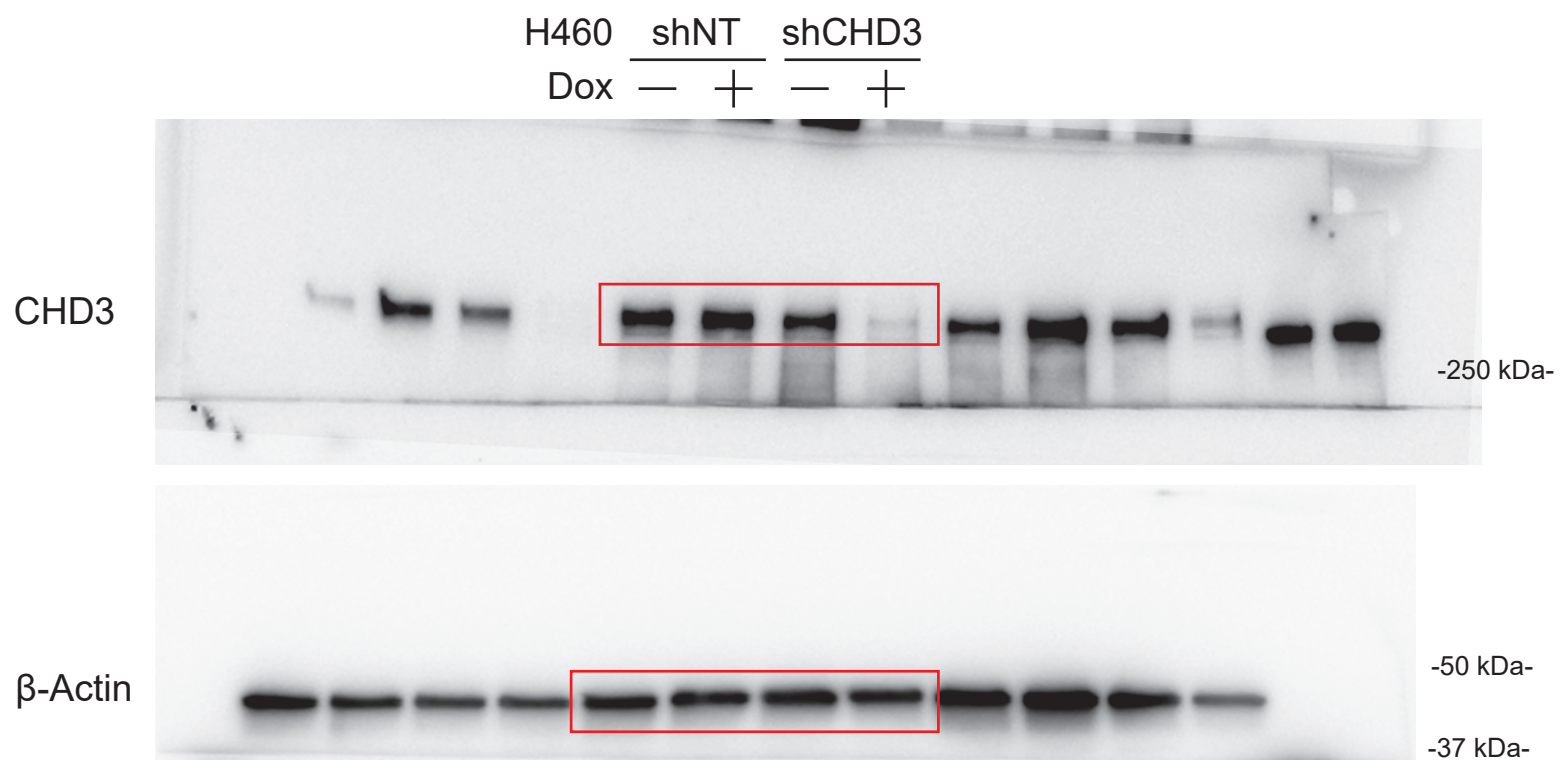

I

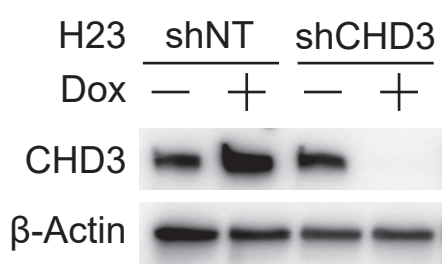

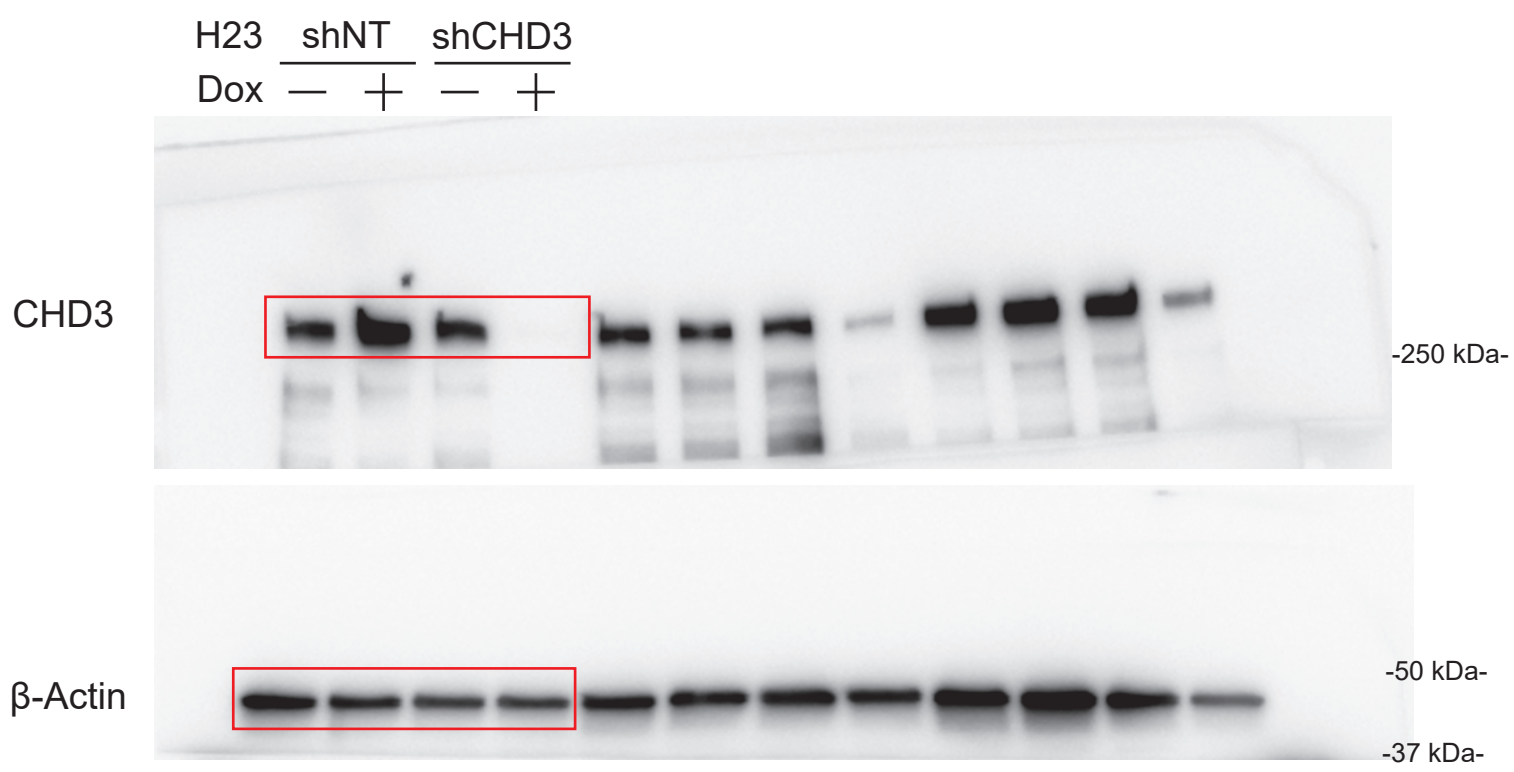

Figure 2

E

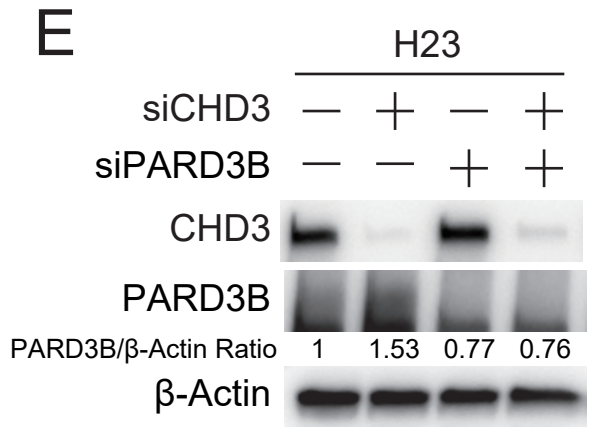

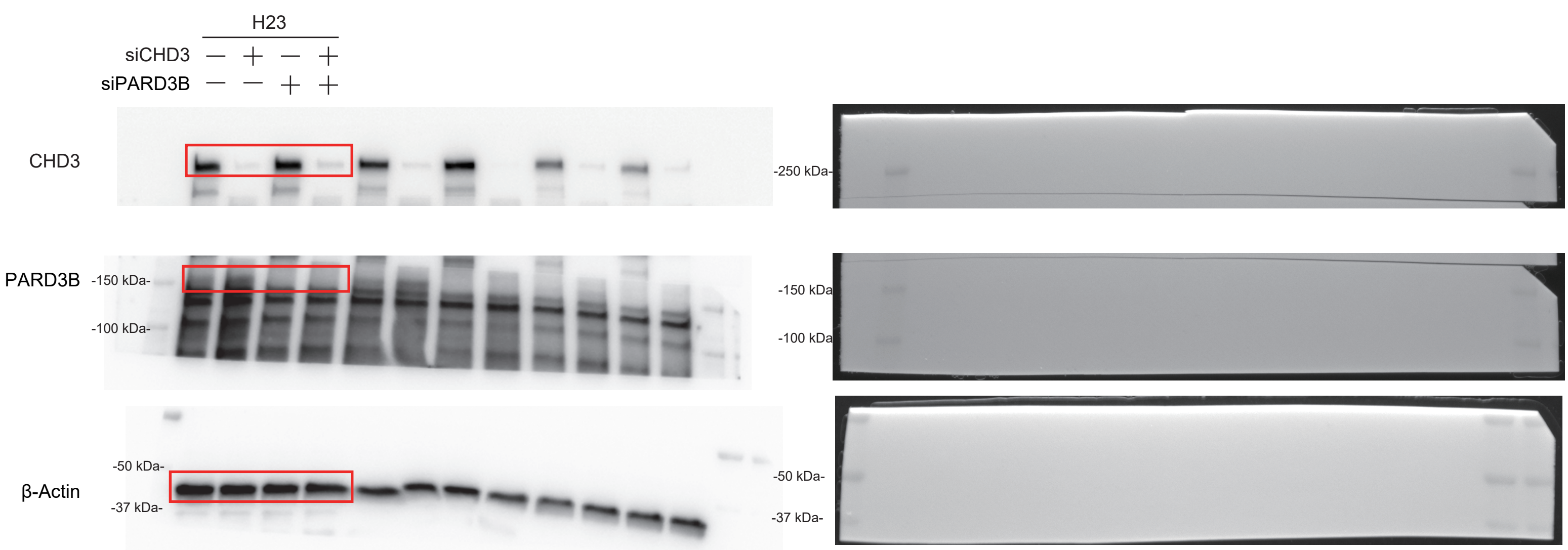

Figure 2

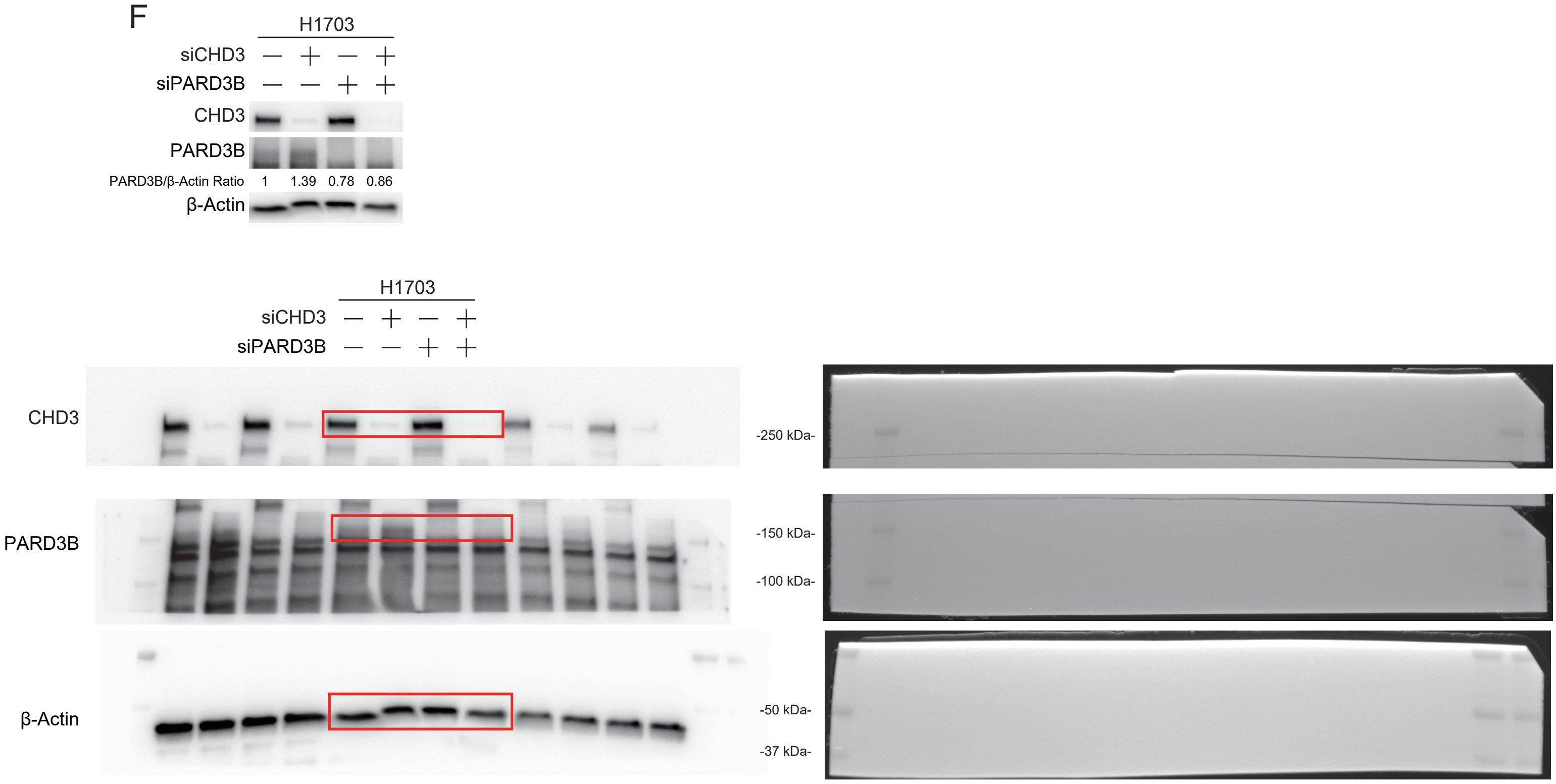

Figure 4

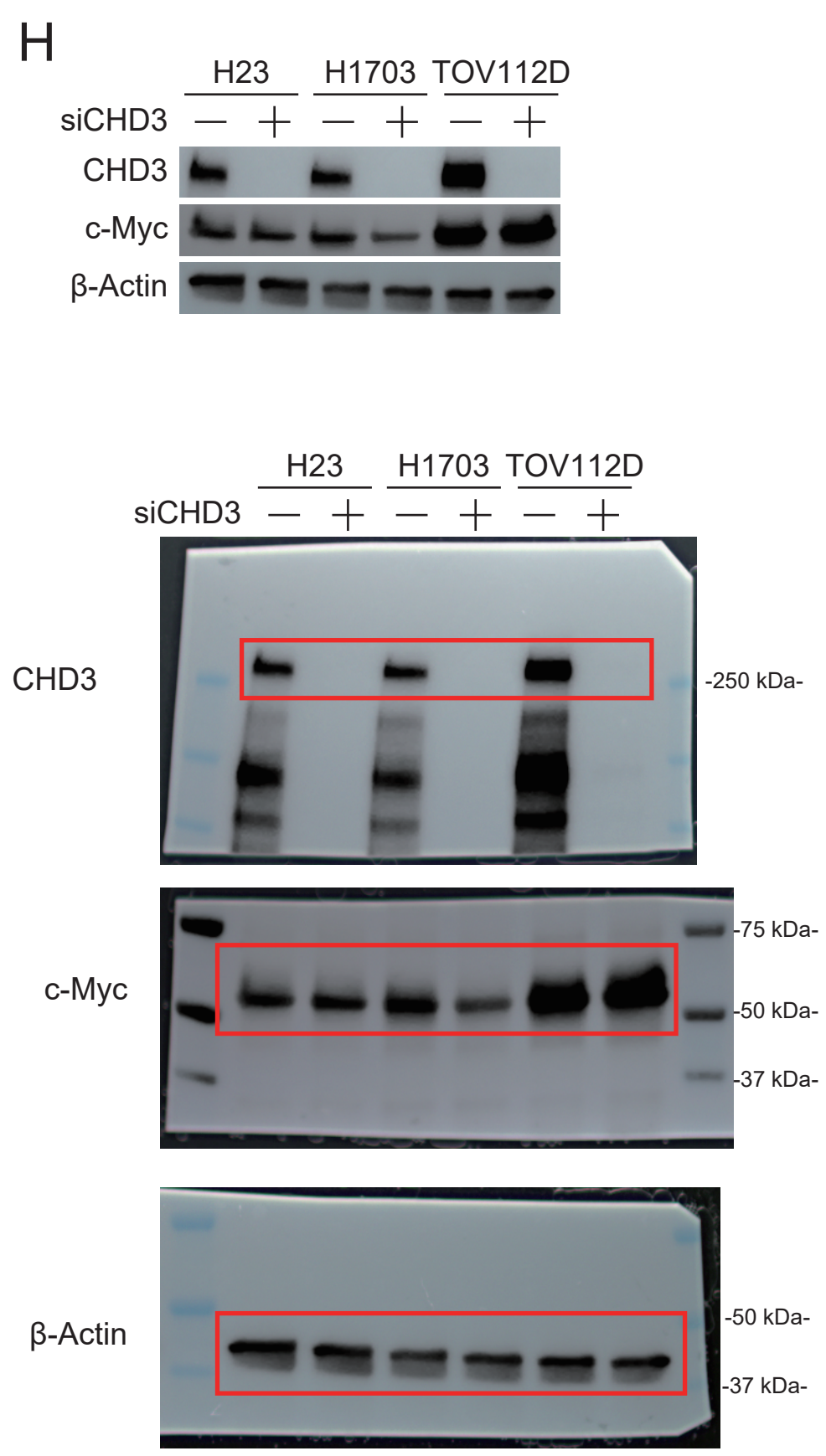

Figure 4

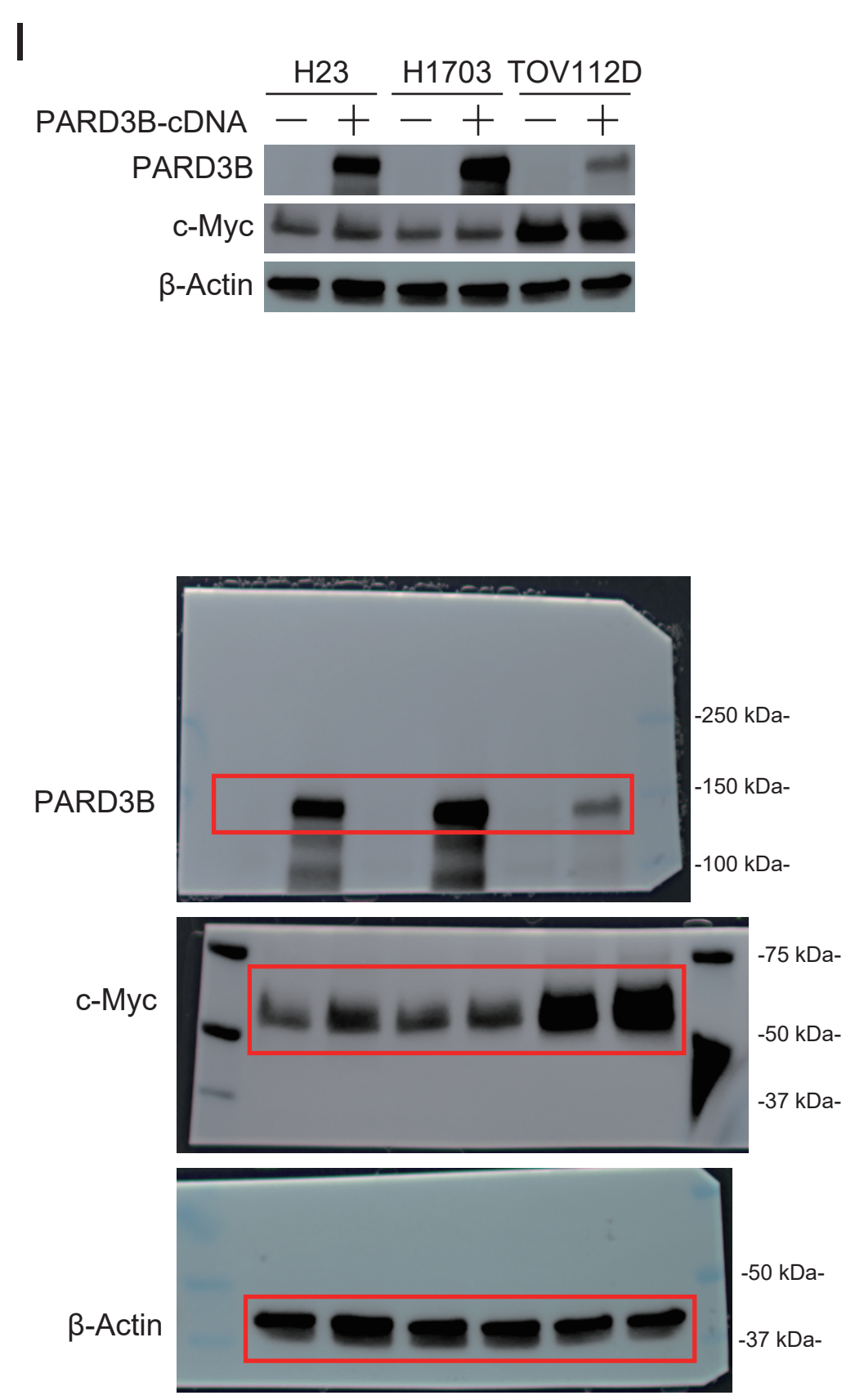

Supplementary Figure 4

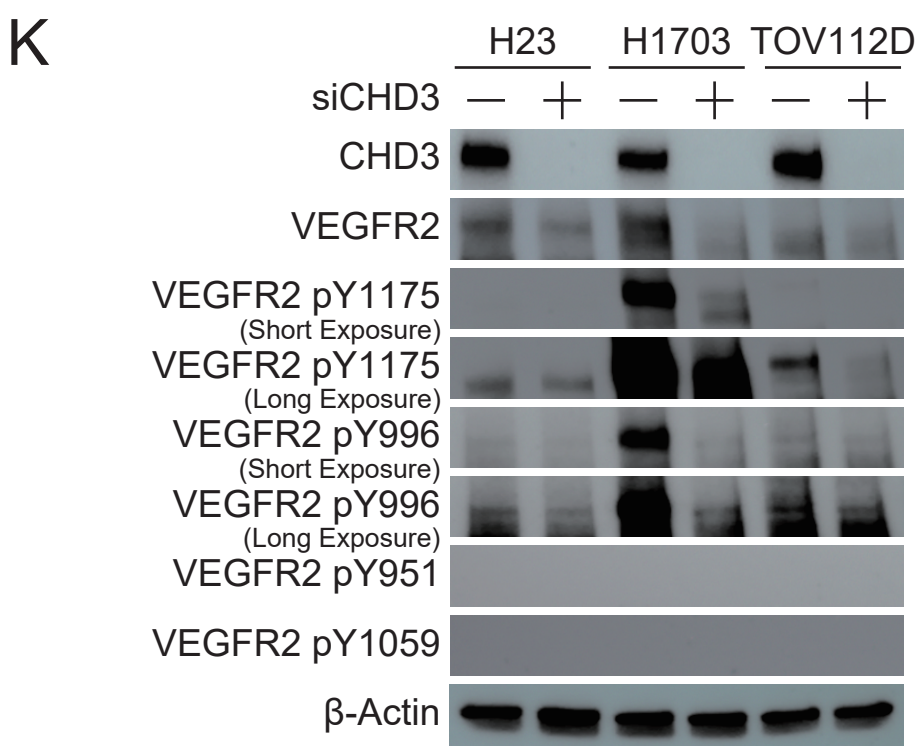

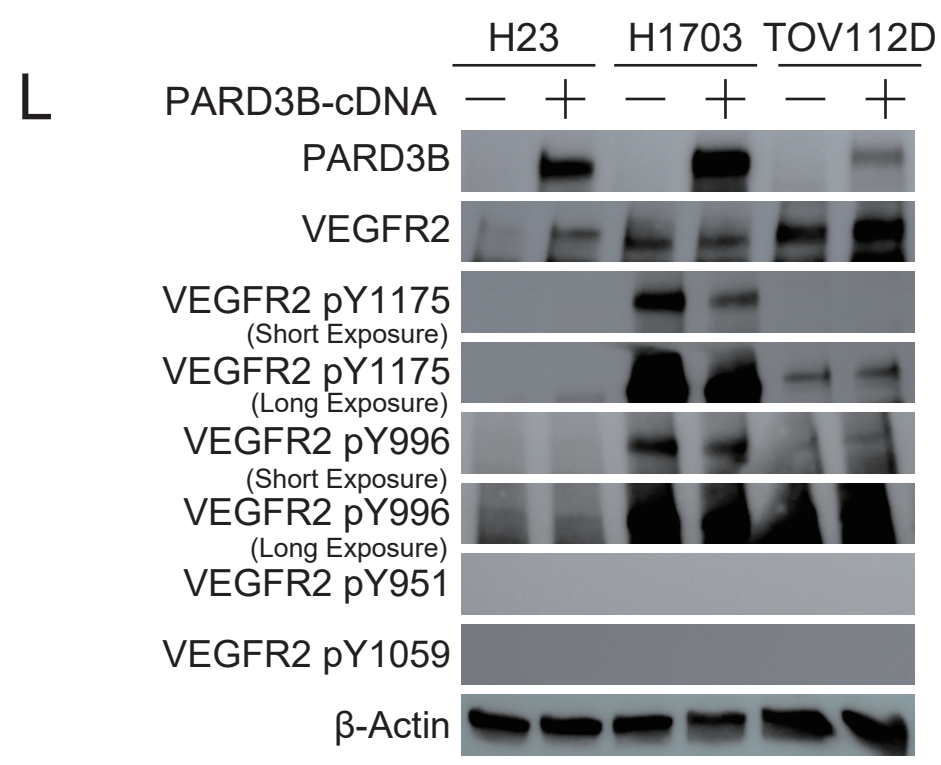

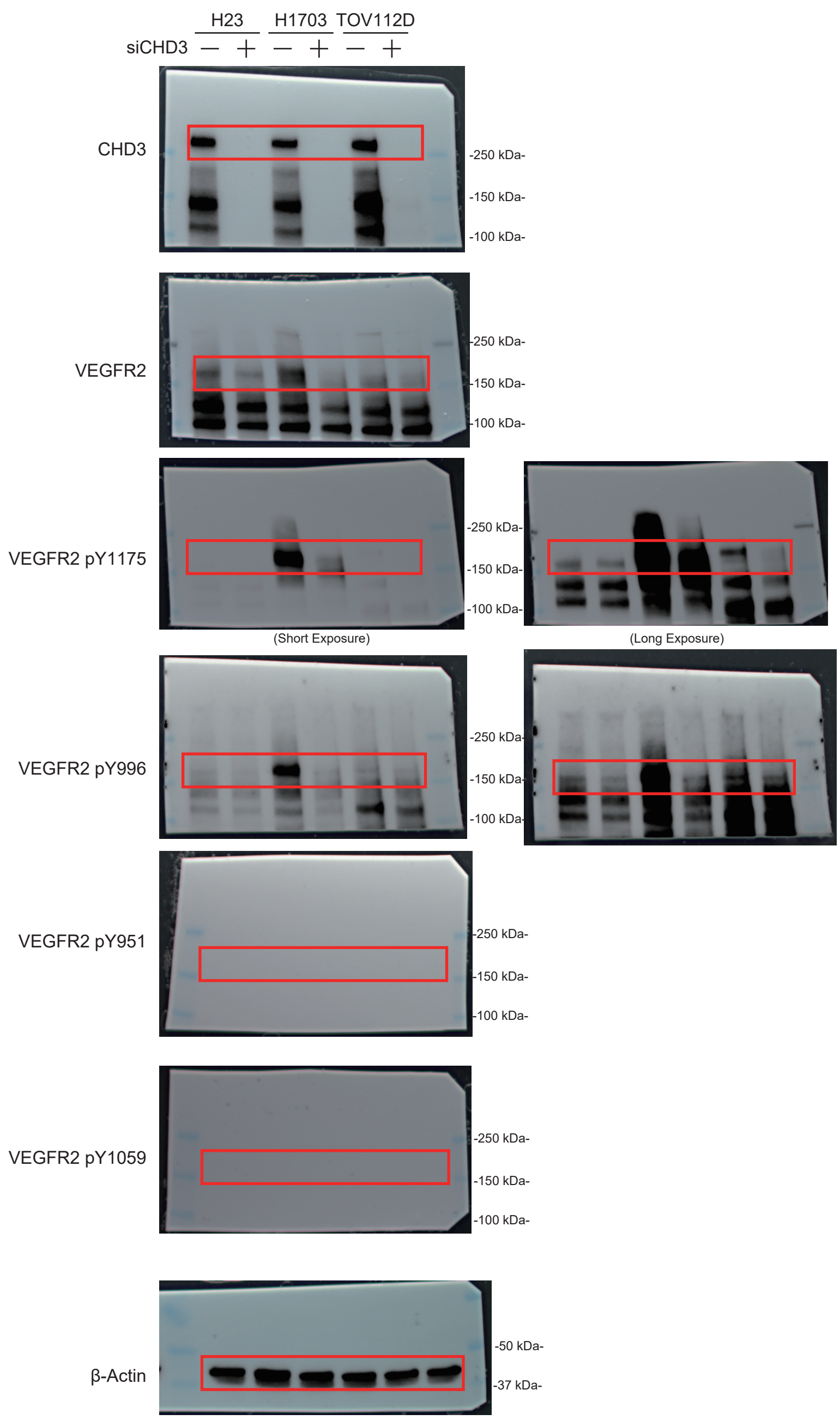

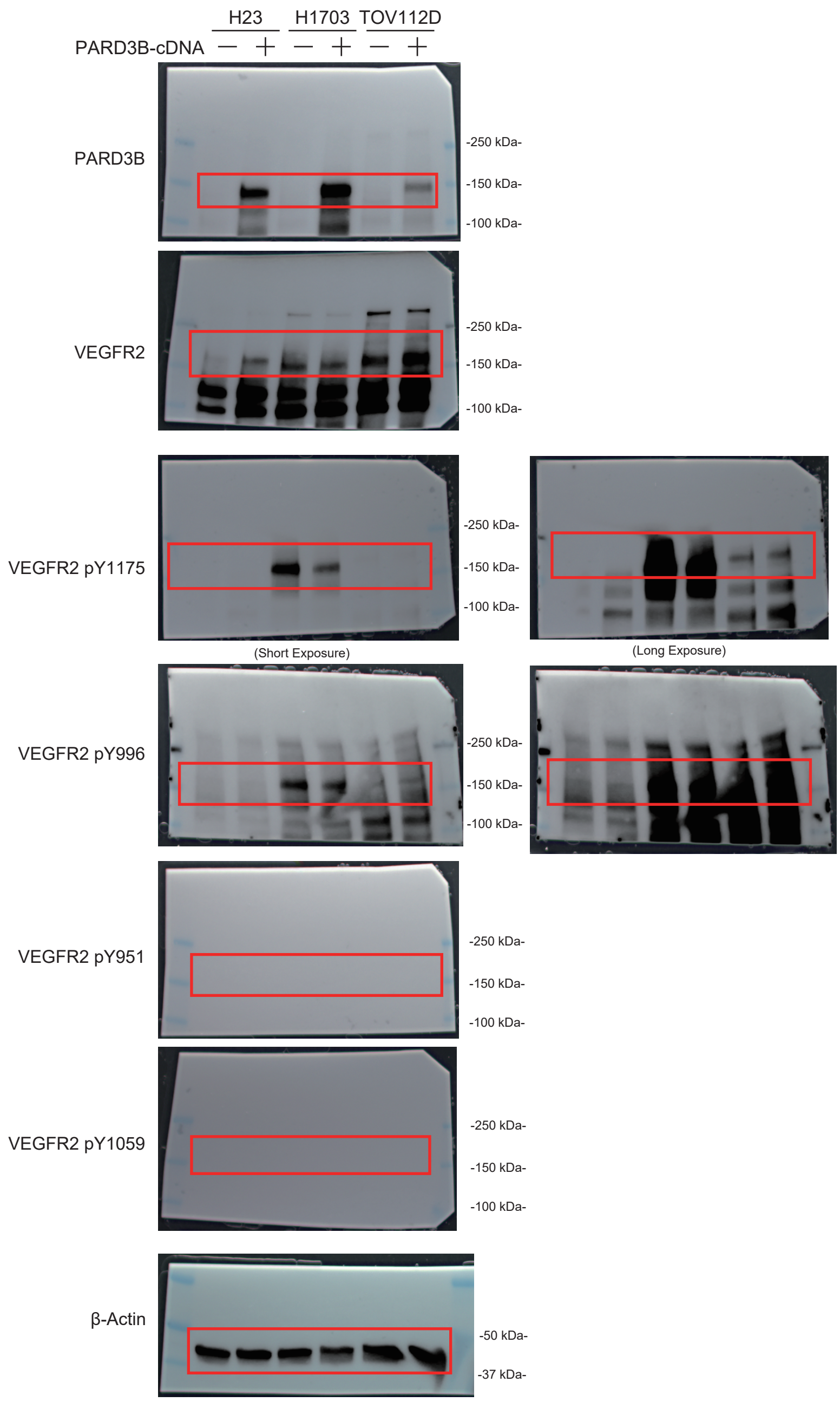

Supplementary Figure 4

N

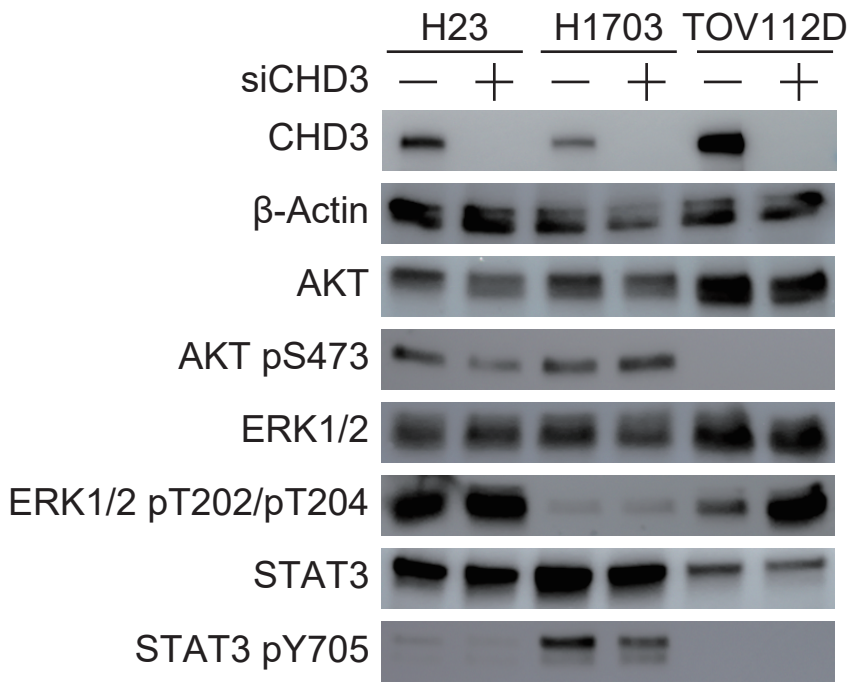

O

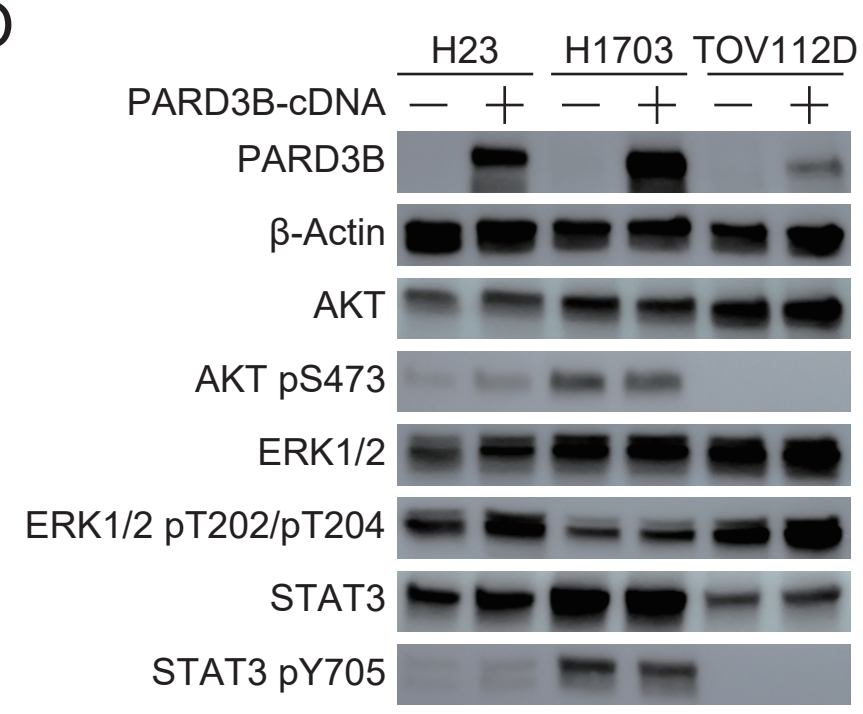

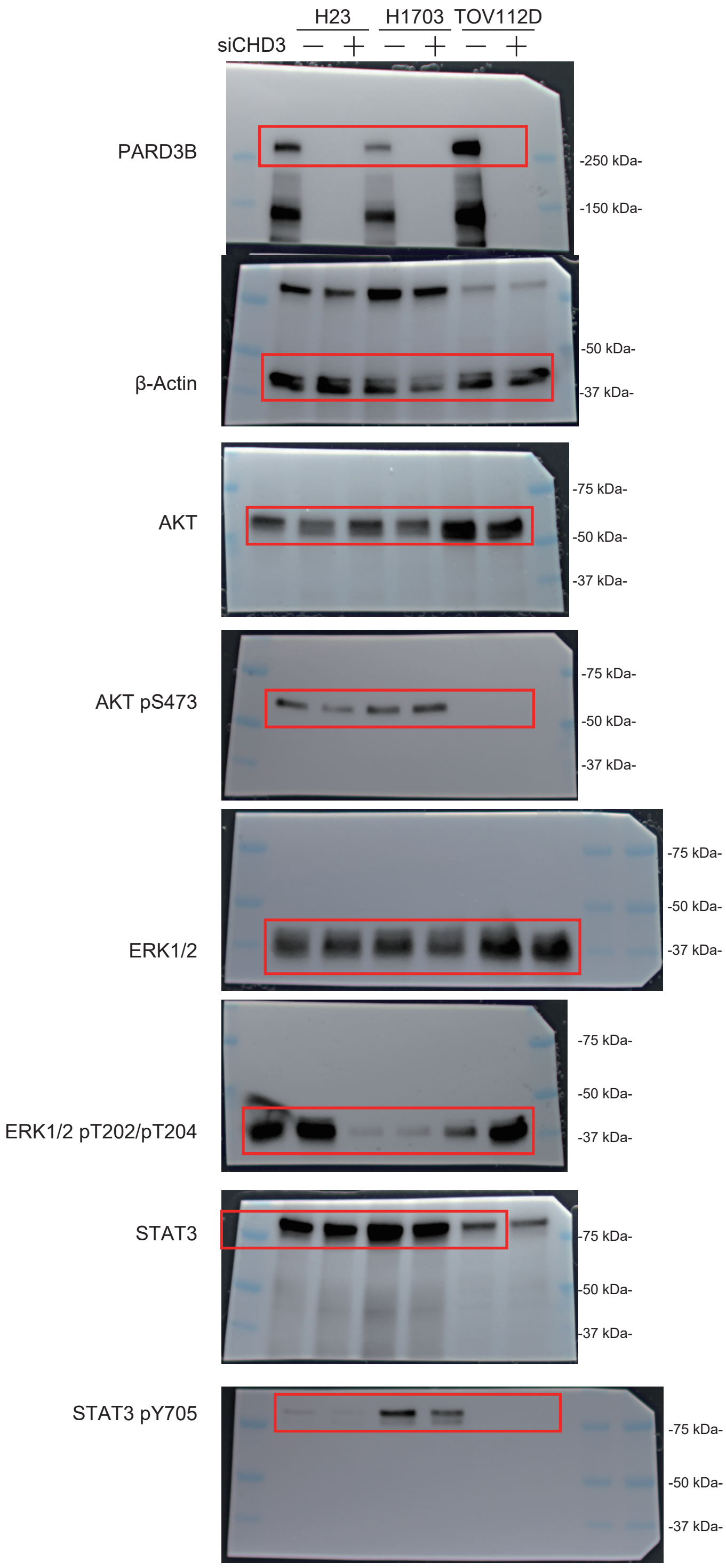

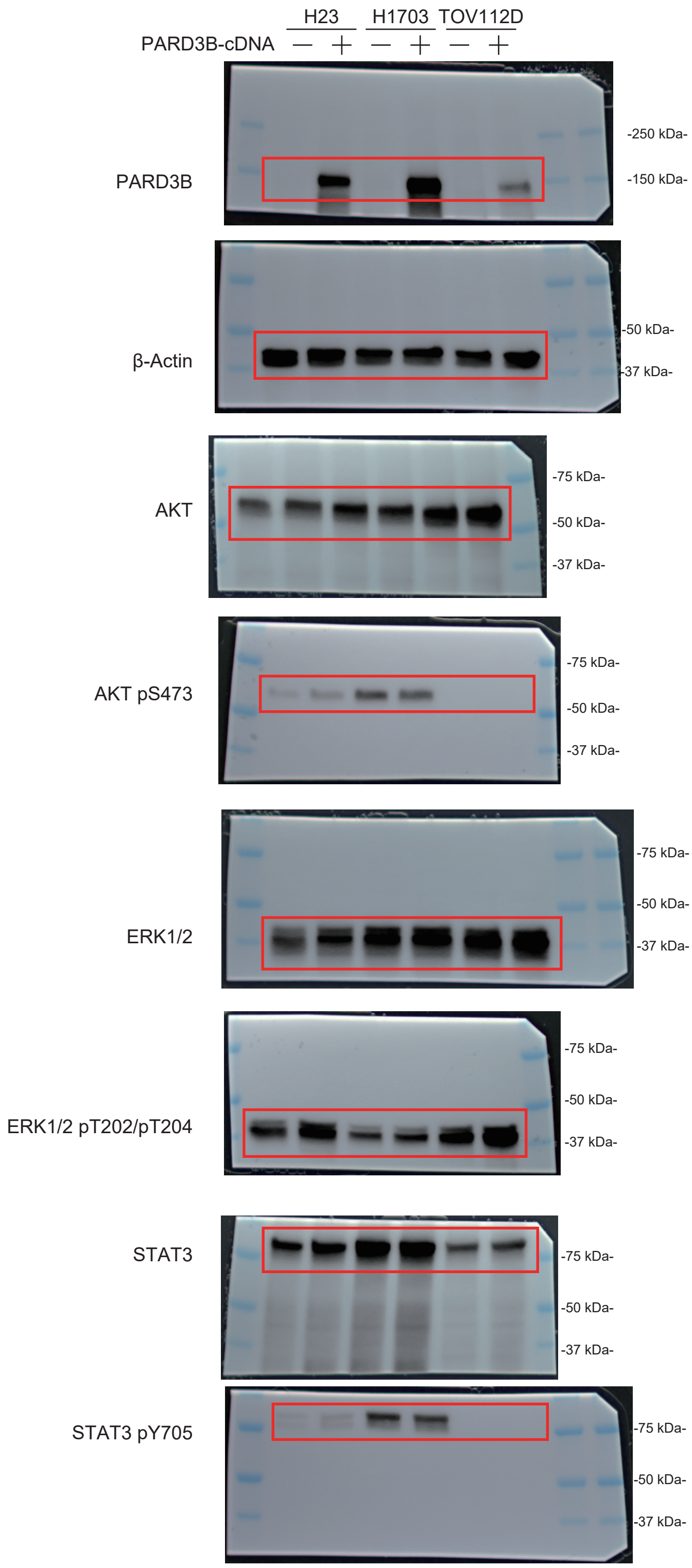

Figure 5

C

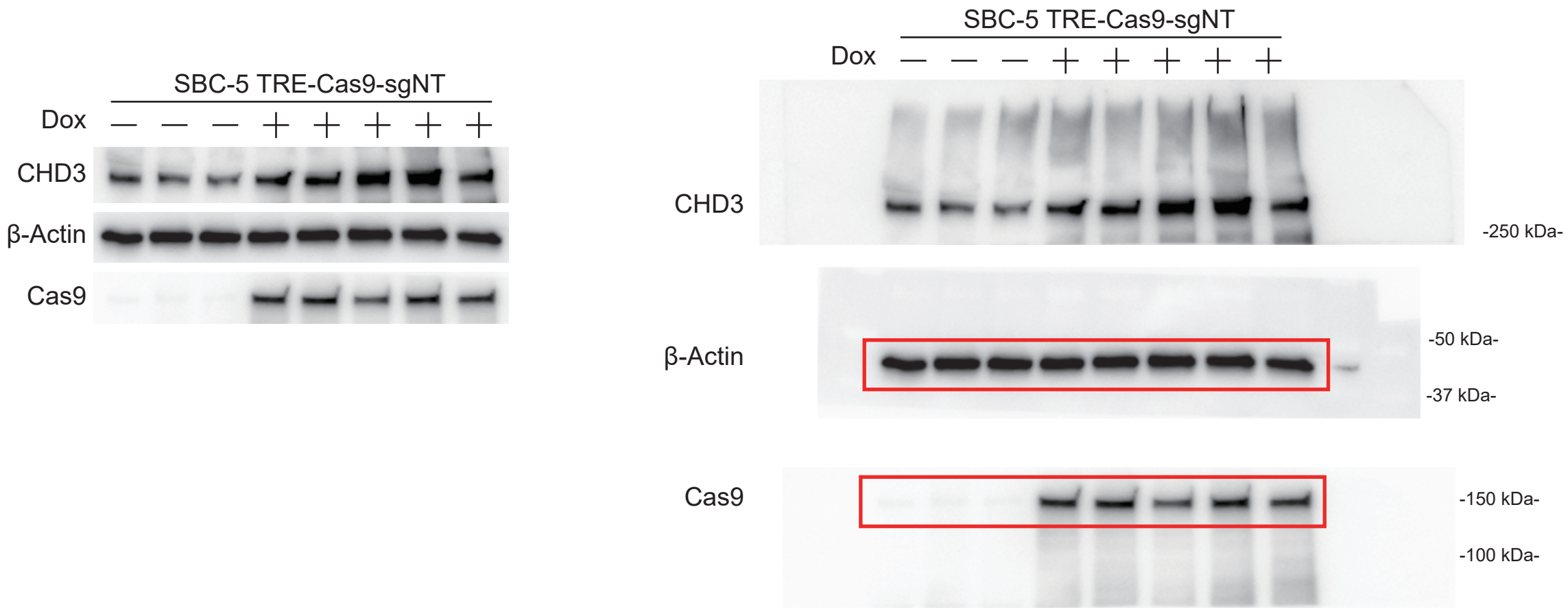
SBC-5 TRE-Cas9-sgNT
Dox — — — + + + + +
CHD3
β-Actin
Cas9
SBC-5 TRE-Cas9-sgNT
Dox — — — + + + + +
CHD3
-250 kDa-
β-Actin
-50 kDa-
-37 kDa-
Cas9
-150 kDa-
-100 kDa-

G

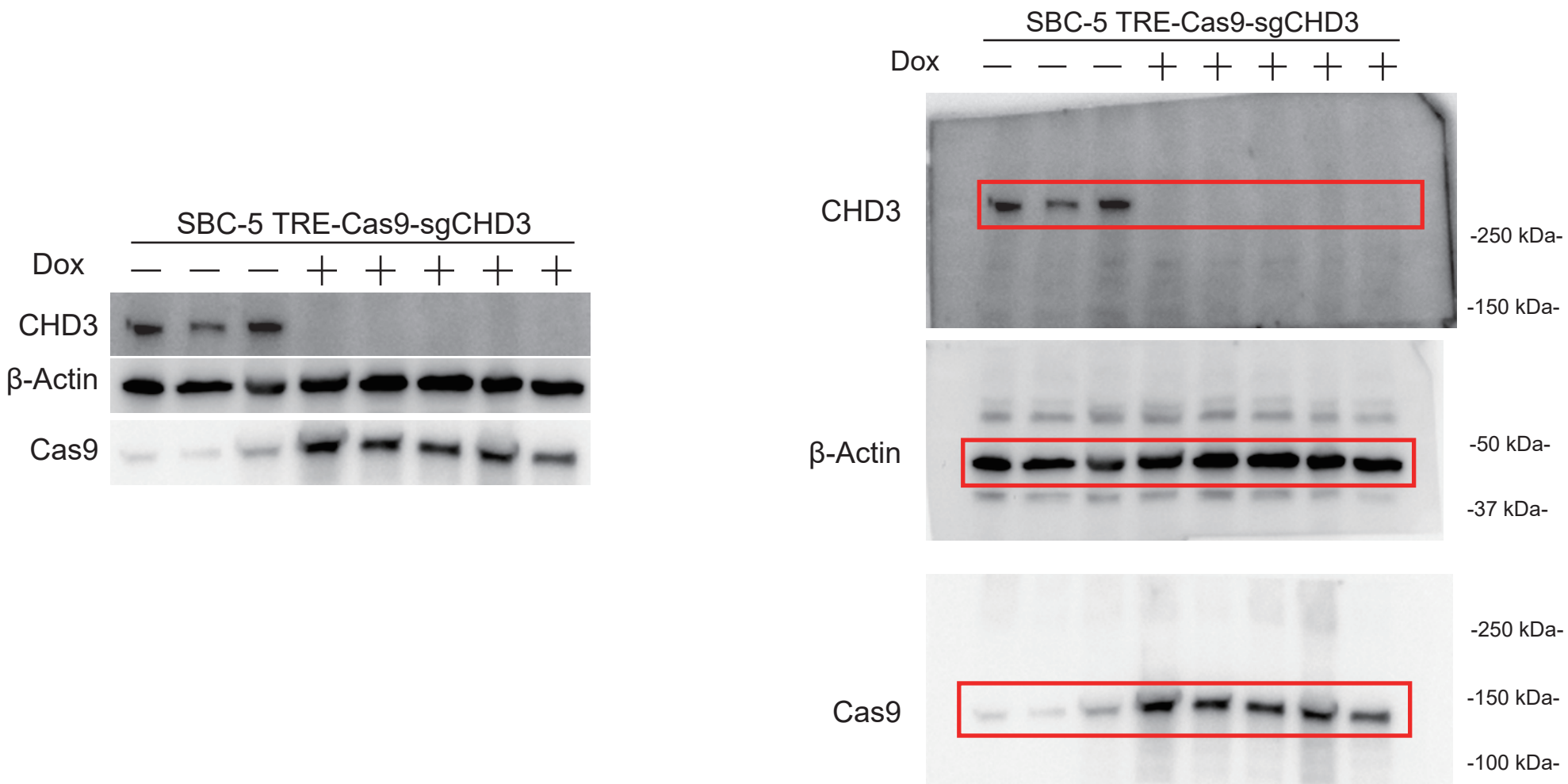
SBC-5 TRE-Cas9-sgCHD3
Dox — — — + + + + +
CHD3
β-Actin
Cas9
SBC-5 TRE-Cas9-sgCHD3
Dox — — — + + + + +
CHD3
-250 kDa-
-150 kDa-
β-Actin
-50 kDa-
-37 kDa-
Cas9
-250 kDa-
-150 kDa-
-100 kDa-
